# Biogenic Selenium Nanoparticles from *Lactiplantibacillus plantarum* as a Potent Antimicrobial Agent Against Methicillin-Resistant *Staphylococcus aureus*

**DOI:** 10.3390/pharmaceutics18010014

**Published:** 2025-12-22

**Authors:** Gyeong-min Kim, SeCheol Oh, Kwang-sun Kim

**Affiliations:** Department of Chemistry and Chemistry Institute for Functional Materials, Pusan National University, Busan 46241, Republic of Korea; eoxhdfud1026@pusan.ac.kr (G.-m.K.); ohs@pusan.ac.kr (S.O.)

**Keywords:** selenium nanoparticles, biosynthesis, *Lactiplantibacillus plantarum* subsp. *plantarum*, methicillin-resistant *Staphylococcus aureus*, synergistic antibiotics, cell-free supernatant

## Abstract

**Background:** Methicillin-resistant *Staphylococcus aureus* (MRSA) remains a major global health concern owing to its multidrug resistance and persistence despite continued antibiotic development. Eco-friendly nanomaterials such as selenium nanoparticles (SeNPs) have emerged as promising antimicrobial alternatives because of their high biocompatibility and lower toxicity compared to conventional metallic nanoparticles. In this study, we investigated the inhibitory effects and underlying mechanisms of *Lactiplantibacillus plantarum* (LP)–derived SeNPs (LP-SeNPs) on MRSA. **Methods:** SeNPs were biosynthesized using the antibacterial cell-free supernatant (CFS) of LP, which provides naturally reducing and stabilizing biomolecules. The resulting LP-SeNPs were characterized by physicochemical and structural analyses and compared to chemically synthesized SeNPs (Chem-SeNPs). Antibacterial activity was assessed through minimum inhibitory concentration (MIC) testing, time-kill kinetics, and cell viability assays. **Results:** LP-SeNPs, which were spherical with an average diameter of 107 nm, exhibited selective antibacterial activity against Gram-positive bacteria and showed no effect on Gram-negative strains. Notably, all six MRSA isolates demonstrated high susceptibility, with MIC values approximately 100-fold lower than that of *S. aureus* ATCC 25923, a non-MRSA reference strain. LP-SeNPs were also non-cytotoxic up to 20-fold the MIC (IC_50_ > 10 µg/mL). Mechanistic analyses indicated that disruption of the bacterial cell membrane was the primary antibacterial mechanism, supported by additional contributions from reactive oxygen species generation and protein synthesis inhibition. **Conclusions:** LP-SeNPs represent a sustainable, biocompatible, and potent antibacterial nanoplatform with strong selectivity for Gram-positive pathogens, particularly MRSA. These findings highlight their potential as eco-friendly and targeted therapeutic strategies for combating MRSA infections.

## 1. Introduction

Methicillin-resistant *Staphylococcus aureus* (MRSA) remains a serious global health threat and is included among the ESKAPE pathogens—*Enterococcus faecium*, *S. aureus*, *Klebsiella pneumoniae*, *Acinetobacter baumannii*, *Pseudomonas aeruginosa*, and *Enterobacter* spp., identified by the World Health Organization as priority pathogens because of their exceptional ability to evade conventional antimicrobial therapies [1]. The increasing prevalence of multidrug-resistant (MDR) MRSA strains, which exhibit resistance to several antibiotic classes including last-line agents such as vancomycin and linezolid, has rendered clinical management increasingly difficult [2,3]. MDR MRSA infections are associated with prolonged disease progression, elevated mortality, and poor clinical outcomes compared to infections caused by susceptible strains [4]. Despite ongoing antibiotic development, MRSA infection rates remain alarmingly high, underscoring the urgent need for alternative therapeutic strategies [5].

Metallic nanoparticles (NPs) have emerged as promising antibacterial candidates because of their broad-spectrum activity, inherent physicochemical versatility, and ability to circumvent traditional resistance mechanisms [6]. However, their clinical translation has been hindered by concerns regarding toxicity, environmental accumulation, and regulatory barriers. For example, silver and gold NPs—among the most widely investigated—can induce significant cellular damage, with toxicity influenced by particle size, morphology, and surface chemistry [7,8,9]. These limitations have intensified interest in biocompatible and eco-friendly nanomaterials produced through green synthesis methods, biopolymer coatings, or synergistic NPs–antibiotic combinations [10,11].

Selenium NPs (SeNPs), derived from an essential micronutrient, offer a safer and more biologically compatible alternative to conventional metallic NPs [12]. Unlike toxic metals such as silver, zinc, and copper, SeNPs exhibit substantially lower cytotoxicity at antimicrobial concentrations and participate naturally in biogeochemical cycling, reducing risks of environmental persistence and bioaccumulation [13,14,15].

Biological routes for SeNPs synthesis—using plant extracts, polysaccharides, or proteins—provide sustainable alternatives to chemically driven methods [16]. However, plant-based synthesis often suffers from batch-to-batch variation owing to fluctuating metabolite profiles [17], and protein-mediated synthesis can be limited by instability, purification challenges, and scale-up difficulties [18]. In contrast, microbial synthesis offers controlled fermentation parameters, reproducible metabolic output, and consistent NPs production [19], establishing microbial platforms as powerful and scalable systems for SeNP biosynthesis.

Among the microbial candidates, lactic acid bacteria (LAB), broadly recognized as Generally Recognized As Safe (GRAS), possess several advantages. LAB secrete abundant and stable biomolecules, including proteins and polysaccharides—that function as natural reducing and capping agents, enabling the formation of uniform and environmentally benign SeNPs. Their fermentative metabolism also allows for precise regulation of culture conditions, thereby enhancing reproducibility. Among LAB, *Lactiplantibacillus plantarum* (LP) is particularly notable for its metabolic flexibility and its strong intrinsic antimicrobial activity. LP produces bioactive metabolites in cell-free supernatants (CFSs) that disrupt MRSA cell integrity [20] and demonstrates exceptional efficiency in reducing Se(VI) from selenite (SeO_3_^2−^) to elemental Se (Se^0^) [21], making it an ideal microbial chassis for SeNPs biosynthesis.

Recent studies have explored the intracellular biotransformation of Se by LP strains. For example, the LP strain BSe was shown to reduce Se(IV) to Se^0^, forming SeNPs with antibacterial activity against *Escherichia coli* and *S. aureus* [22]. However, intracellular methods require multiple cell lysis and purification steps, rely on harsh chemicals, and leave the antibacterial efficacy and mechanism insufficiently characterized. Conversely, extracellular synthesis using LAB-derived CFS (LAB-CFS) offers a cleaner, more scalable, and more environmentally friendly alternative. LAB-CFS contains natural reducing and capping agents capable of converting toxic inorganic Se into biogenic SeNPs with improved biological performance and reduced toxicity [21]. To address the limitations of intracellular biotransformation [22] and fully leverage the synergistic potential of LP and SeNPs, we employed LP-derived CFS (LP-CFS) to drive extracellular SeNPs biosynthesis and systematically characterized the resulting NPs.

In this study, we report the successful synthesis and comprehensive characterization of LP-CFS-assisted SeNPs (LP-SeNPs). The reaction yielded a distinct reddish pellet of LP-SeNPs, producing approximately 0.7 µg per mL of LP-CFS. The physicochemical properties of the LP-SeNPs were characterized using Fourier-transform infrared spectroscopy (FTIR), X-ray diffraction (XRD), transmission electron microscopy (TEM), X-ray photoelectron spectroscopy (XPS), zeta potential analysis, UV–Visible (UV-Vis) spectroscopy, and dynamic light scattering (DLS), and directly compared with chemically synthesized SeNPs (Chem-SeNPs). Antibacterial activity was evaluated using minimum inhibitory concentration (MIC) determination, time-kill curve assays, and viability spotting analyses. LP-SeNPs exhibited markedly enhanced antibacterial activity against all tested MRSA strains while maintaining minimal cytotoxicity toward two mammalian cell lines—even at concentrations 20-fold higher than the MIC. Mechanistic studies—including reactive oxygen species (ROS) generation assays, scanning electron microscopy (SEM)-based morphological analysis, total cellular protein quantification, and synergistic antibiotic interaction testing—consistently indicated that membrane disruption is a central mechanism underlying LP-SeNPs–mediated MRSA killing. Overall, these findings establish LP-SeNPs as a potent, eco-friendly, and biocompatible antimicrobial platform with a strong potential for combating MDR MRSA infections.

## 2. Materials and Methods

### 2.1. Bacterial Strains

The non-MDR bacterial strains used in this study were obtained from the American Type Culture Collection (ATCC; https://www.atcc.org, Manassas, VA, USA) and the National Culture Collection for Pathogens (NCCP; https://nccp.kdca.go.kr/). Clinical MRSA isolates previously characterized in an earlier study [23] were also used.

### 2.2. Synthesis of LP-SeNPs and Chem-SeNPs

**Figure 1:**
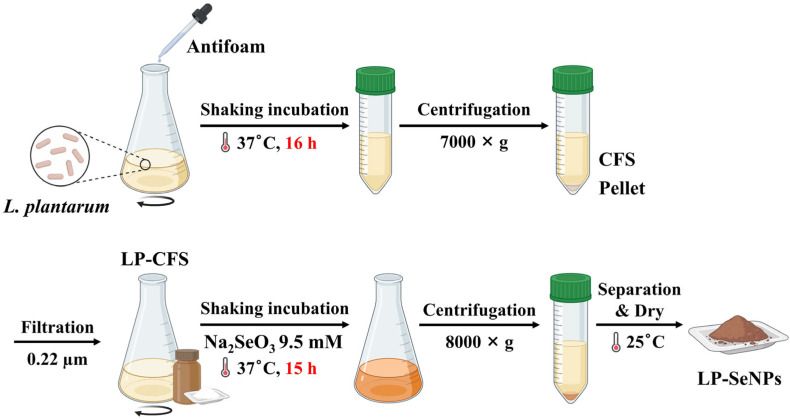
Schematic representation of SeNPs synthesis using LP-derived CFS (LP-CFS). This method utilizes LP-CFS, which is free from cells, cellular debris, and chemical reagents, enabling the green synthesis of LP-SeNPs.

A schematic overview of LP-SeNPs synthesis is presented in Figure 1. LP (ATCC 14917) was sub-cultured in de Man, Rogosa, and Sharpe (MRS) broth (BD Diagnostic Systems, Sparks, MD, USA) under aerobic conditions at 37 °C for 9 h and then incubated for an additional 16 h with 20 µL of Antifoam A concentrate (0.0001% final concentration; Sigma-Aldrich, St. Louis, MO, USA) to minimize oxidation during shaking. The culture was centrifuged at 6000× *g* for 30 min to obtain LP-CFS, which was filtered through a 0.22 µm syringe filter (BioFact, Daejeon, Republic of Korea).

Extracellular SeNPs synthesis was initiated by adding sodium selenite (Na_2_SeO_3_; Sigma-Aldrich) to filtered LP-CFS at a final concentration of 9.5 mM in 100 mL. The mixture was then incubated at 37 °C with shaking for 15 h. The resulting LP-SeNPs were harvested by centrifugation at 7800× *g* for 30 min, washed thoroughly with deionized distilled water (DDW), dried at 25 °C for 24 h, and stored in sterile vials.

Chem-SeNPs were produced following a modified version of a previously described method [24]. Briefly, 30 mg of Na_2_SeO_3_ was dissolved in 90 mL of DDW, and 10 mL of ascorbic acid (56.7 mM) was added dropwise under vigorous stirring. During reduction, 10 µL of polysorbate was added after every 2 mL of ascorbic acid to enhance the NP stability. The formation of Chem-SeNPs was confirmed by the rapid color change from colorless to reddish-orange. NPs were collected by centrifugation at 13,000× *g* for 30 min, washed with DDW, dried at 25 °C for 24 h, and stored in sterile vials for further analysis.

### 2.3. Characterization of LP-SeNPs and Chem-SeNPs

LP-SeNPs and Chem-SeNPs were characterized using established analytical methods [25,26]. The FTIR spectra of MRS broth, LP-CFS, and LP-SeNPs were recorded using an Agilent Cary 630 FTIR spectrometer (Agilent Technologies, Santa Clara, CA, USA). XRD patterns of both SeNP types were acquired on a D8 Advance diffractometer (Bruker Nano GmbH, Berlin, Germany) equipped with Ni-filtered Cu Kα radiation (λ = 1.5406 Å) over a 2θ range of 20–80°. TEM analysis (Bruker Nano GmbH) was performed on carbon-coated 300-mesh Cu grids after sonication for 10 min in DDW. XPS spectra were obtained using an AXIS Supra instrument (Kratos Analytical, Manchester, UK) to examine surface chemical states. The hydrodynamic size distribution and zeta potential were measured using a nanoPartica SZ-100-S2 (Horiba Ltd., Kyoto, Japan). UV–Vis spectra were collected using a UV-2600i spectrophotometer (Shimadzu, Kyoto, Japan). Representative results from triplicate experiments are shown with the values of mean ± standard deviation (SD).

### 2.4. Analysis of Antibacterial Activity

#### 2.4.1. MIC Determination

MIC values were determined using a 96-well microdilution assay, with minor modifications to standard methods [27]. Bacterial colonies from LB agar were resuspended in DDW, adjusted to a 0.5 McFarland standard (Sensititre™ Nephelometer; Thermo Fisher Scientific), and diluted 1:1000 in cation-adjusted MHB (KisanBio, Seoul, Republic of Korea). Chem-SeNPs and LP-SeNPs (1 mg/mL stocks) were serially diluted to 0–100 µg/mL concentrations. Each dilution (20 µL) was added to 180 µL of bacterial suspension in 96-well plates (SPL Life Sciences, Pocheon, Republic of Korea). The plates were incubated at 37 °C without shaking for 16 h. Images of the 96-well plates were captured using a digital camera (Samsung NX200, Suwon, Republic of Korea). All MIC values indicated in this study were identical across three independent replicates and, therefore, were reported as fixed values rather than mean ± SD.

#### 2.4.2. Synergy Testing with Conventional Antibiotics

Synergistic activity between LP-SeNPs at one-quarter of their MIC (¼ MIC) and selected antibiotics—colistin (COL), erythromycin (ERY), imipenem (IMP), rifampin (RIF), and tetracycline (TET)—at concentrations ranging from 0 to 64 µg/mL was evaluated against MRSA strains. Fractional inhibitory concentration indices (FICI) using MIC values from assays performed with or without LP-SeNPs at ¼ MIC were calculated as follows [28]:

FICI = (MIC of LP-SeNPs in combination/MIC of LP-SeNPs alone) + (MIC of antibiotic in combination/MIC of antibiotic alone).

Synergy and no synergy are defined as FICI ≤ 0.5 and FICI > 0.5, respectively [29,30]. Representative results from triplicate experiments are shown. Images of the 96-well plates were captured using a digital camera (Samsung NX200, Suwon, Republic of Korea).

#### 2.4.3. Colony Forming Unit (CFU) Determination

The antibacterial activity of LP-CFS is described in Section 2.2. were assessed by CFU enumeration. MRSA4, as a representative of MRSA isolates, cell suspensions in DDW were adjusted to 0.5 McFarland, inoculated into MHB, and grown to OD_600_ = 1.0. LP-CFS treatment consisted of mixing 300 µL of LP-CFS with 2.7 mL of MHB and 50 µL of culture; the controls used 300 µL DDW. After 3 h of incubation at 37 °C with shaking, the suspensions were diluted (10^−5^), plated onto MH agar, and incubated for 16 h. Plates were imaged using a ChemiDoc^TM^ MP Imaging System (Bio-Rad, Hercules, CA, USA) and processed with Image Lab^TM^ software (v5.2.1, Bio-Rad). The CFUs were manually counted. The percentage of growth inhibition by LP-CFS was calculated as follows:

Inhibition (%) = 100 − [(CFU of LP-CFS–treated cells)/(CFU of control)] × 100.

Three independent images and the inhibition (%) were indicated. In addition, mean ± SD values are also presented.

#### 2.4.4. Time-Kill Curve Assays

Bacterial suspensions adjusted to 0.5 McFarland as prepared in Section 2.4.1 were diluted 1:1000 in 180 µL MHB and mixed with 20 µL of LP-SeNPs at various concentrations. Plates were incubated at 37 °C with shaking (500 rpm), and OD_600_ was monitored every 30 min for 16 h using SPECTROStar^®^ Nano (BMG Labtech, Ortenberg, Germany). Data were processed using the MARS software (v3.02 R2; BMG Labtech). For endpoint viability, 5 µL of each culture was plated on LB agar and incubated for 16 h. Plate images were acquired using a ChemiDoc^TM^ MP Imaging System (Bio-Rad) and processed using Image Lab^TM^ software (v5.2.1, Bio-Rad). Representative results from triplicate experiments are shown.

### 2.5. In Vitro Cytotoxicity Assay

The in vitro cytotoxicity of LP-SeNPs toward HeLa and human embryonic kidney (HEK)-293T cells was evaluated using the 3-(4,5-dimethylthiazol-2-yl)-2,5-diphenyltetrazolium bromide (MTT) assay, with minor modifications [31]. Cells were cultured in Dulbecco’s Modified Eagle’s medium (DMEM; Cytiva, Marlborough, MA, USA) supplemented with 10% fetal bovine serum (FBS) and seeded at a density of ~5 × 10^4^ cells/mL. in 96-well microplates (cat. No. 30096; SPL Life Sciences) and treated with varying concentrations of LP-SeNPs or phosphate-buffered saline (PBS, pH7; control). Plates were incubated at 37 °C with 5% CO_2_ for 24 and 48 h. Following incubation, MTT solution (0.5 mg/mL) was added to each well, and the plates were incubated for 4 h at 37 °C under 5% CO_2_. The absorbance at 595 nm (A_595_) was measured using a SPECTROStar^®^ Nano spectrophotometer (BMG Labtech). Relative cytotoxicity is indicated as the ratio of the A_595_ of the treated samples to that of the PBS control. For cytotoxicity assessment, the half-maximal inhibitory concentration (IC_50_) was determined from the dose–response curve. All data are expressed as the mean ± SD from triplicate experiments.

### 2.6. SEM Image Analysis of MRSA

Morphological alterations in MRSA cells treated with LP-SeNPs at ½ MIC were examined by SEM. Cell pellets were harvested at 13,500× *g* for 1 min, fixed in 2% formaldehyde/1% glutaraldehyde for 5 min, washed with DDW, and resuspended in 1 mL of DDW. For the SEM image analysis, 5 µL of the suspension was placed on a silicon wafer and air-dried at 25 °C. Morphological observations were performed using a JSM-IT210 scanning electron microscope (JEOL, Ltd., Tokyo, Japan). The data are representative of triplicate experiments.

### 2.7. Quantification of Total Cellular Proteins

Total cellular protein content of MRSA isolates treated with or without LP-SeNPs was analyzed using 12% TGX stain-free sodium dodecyl sulfate–polyacrylamide (SDS-PAGE) gels (Bio-Rad) and visualized with a ChemiDoc^TM^ MP system (Bio-Rad), following a previously described method with minor modifications [23]. Six MRSA isolates were cultured to an OD_600_ of 0.5, treated with LP-SeNPs at 4 × MIC for 2 h at 37 °C, and harvested via centrifugation at 13,500× *g* for 1 min. Cell pellets were resuspended in RIPA lysis buffer and extraction buffer (Thermo Fisher Scientific), with normalization based on cell numbers. Protein profiles and band intensities were quantified using the Image Lab^TM^ Software (v.5.2.1, Bio-Rad). Representative quantification data are expressed as the mean ± SD from triplicate experiments.

### 2.8. Measurement of ROS Production

The ROS production capacity of LP-SeNPs against *S. aureus* ATCC 25923, MRSA4, and MRSA5 was evaluated based on a previously report [32]. Briefly, bacterial cell suspensions (0.5 McFarland turbidity in PBS) were treated with varying concentrations of LP-SeNPs (0–10 μg/mL) in the presence of 2′,7′-dichlorodihydrofluorescein diacetate (DCFH-DA; Sigma-Aldrich) at a final concentration of 30 µM. The cultures were incubated at 37 °C for 3 h with vigorous shaking (500 rpm). ROS levels were quantified using a FLUOstar^®^ Omega microplate reader (BMG Labtech) by measuring fluorescence intensity at excitation (Ex) and emission (Em) wavelengths of 485 and 520 nm, respectively. Bacterial suspensions in PBS without LP-SeNPs were used as controls. Fluorescence data were further analyzed using the MARS Data Analysis software (ver. 3.02 R2; BMG Labtech). All experiments were performed in triplicate, and the relative ROS production of the treated samples is expressed as the mean ± SD from triplicate experiments.

### 2.9. Role of LP-SeNPs–Associated Biomolecules in Antibacterial Activity

To assess the contribution of LP-SeNP-associated biomolecules to antibacterial activity, protein and DNA components were selectively degraded using proteinase K and DNase. LP-SeNPs suspensions were prepared at a concentration of 1 mg/mL and 500 µL of the suspension was aliquoted into microcentrifuge tubes for each treatment condition. Samples were centrifuged at 13,000× *g* for 30 min to remove the supernatant, and the resulting LP-SeNPs pellets were subjected to enzymatic degradation, as described below.

For protein degradation, proteinase K (Biofact; 20 mg/mL) was added to each pellet to achieve a final concentration of 100 µg/mL, in a total reaction volume of 200 µL (diluted in DDW). Samples were incubated at 37 °C for 1 h. For DNA degradation, RQ1 DNase (Promega, Madison, WI, USA; 1000 U/µL) was used. A reaction mixture consisting of 5 µL DNase, 20 µL 10× DNase buffer, and 180 µL DDW (total volume: 200 µL) was added to each pellet, followed by incubation at 37 °C for 30 min. Control samples were prepared using the same procedure, with DDW substituted for the enzyme solutions.

After each enzymatic reaction, 200 µL of 20 mM ethylenediaminetetraacetic acid (EDTA) was added and incubated for 10 min at room temperature to terminate enzymatic activity. All samples were then centrifuged at 13,000× *g* for 30 min, and the LP-SeNPs pellets were washed three times with DDW to remove residual enzymes and reaction components. The antibacterial activity of enzyme-treated and untreated LP-SeNPs was subsequently assessed using the MIC assay described in Section 2.4.1.

### 2.10. Statistical Analysis

All data are presented as the mean ± SD from triplicate experiments unless otherwise indicated. Statistical significance was assessed using one-way ANOVA followed by Dunnett’s multiple comparisons test. All statistical analyses were performed using GraphPad Prism (ver. 10; GraphPad Software, San Diego, CA, USA). Graphs were generated using OriginPro software (ver. 10.0; OriginLab^®^, Northampton, MA, USA). Statistical significance was defined as *p* < 0.05 (*), *p* < 0.01 (**), *p* < 0.001 (***), and *p* < 0.0001 (****).

## 3. Results and Discussion

### 3.1. Synthesis of SeNPs Using LP-CFS

**Figure 2:**
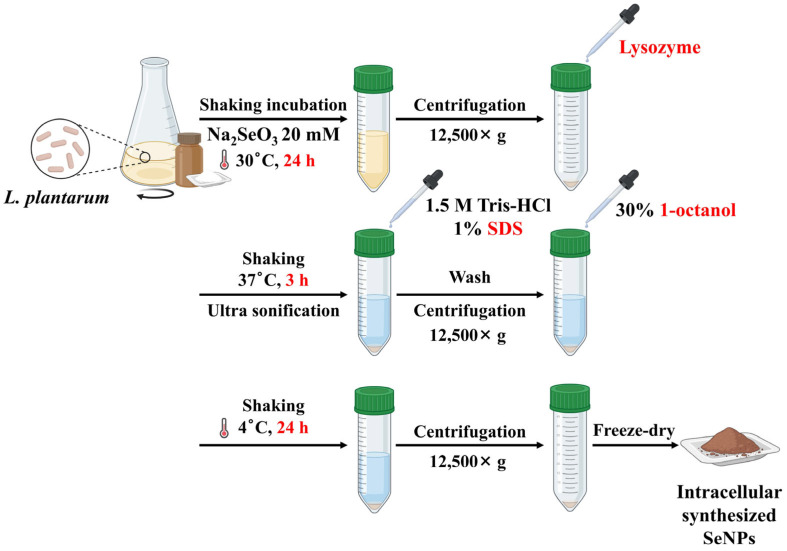
Schematic representation of SeNP synthesis by intracellular biotransformation approach [22].

The previously reported intracellular biotransformation of Se using LP cells [22] presents several critical limitations (Figure 2). Intracellular synthesis requires the addition of Na_2_SeO_3_ directly into MRS cultures containing LP cells, necessitating multiple cell-lysis, extraction, and purification steps to recover SeNPs. This labor-intensive workflow not only reduces yield but also complicates the production of pure SeNPs due to recurrent biological contamination.

Furthermore, the reliance on harsh disruption reagents such as lysozyme, SDS, and 1-octanolraises concerns regarding biocompatibility, safety, and environmental impact. Although lysozyme is generally regarded as safe, it can induce allergic reactions in susceptible individuals [33,34], whereas SDS and 1-octanol are known to cause membrane damage, cytotoxicity, and ecological toxicity [35]. These issues collectively limit the suitability of intracellularly synthesized SeNPs for biomedical and food-grade applications. Importantly, the antibacterial performance of intracellularly derived SeNPs has not been previously validated and no mechanistic investigations have been conducted. As a result, the functional relevance and biological activity of SeNPs remain largely undetermined.

To overcome these limitations while preserving the inherent biosynthetic advantages of LP, we used LP-CFS for SeNPs synthesis (Figure 1). LP-CFS constitutes a cell-free extracellular fraction enriched in naturally occurring reducing and capping biomolecules, providing strong bioreductive activity and excellent biocompatibility [36,37,38]. This biologically active medium enables the efficient conversion of toxic Se(IV) into Se^0^ under mild, non-toxic conditions [21]. Because CFS is obtained through centrifugation and filtration, it is inherently free of cellular debris and intracellular impurities, ensuring a cleaner and more controlled synthesis environment [39].

To further enhance culture performance, a minimal amount of antifoam reagent was added during LP cultivation. This intervention effectively reduced oxidative stress without compromising cell viability, thereby supporting metabolic homeostasis and reinforcing the biotransformation capacity of LP cells [40].

**Figure 3:**
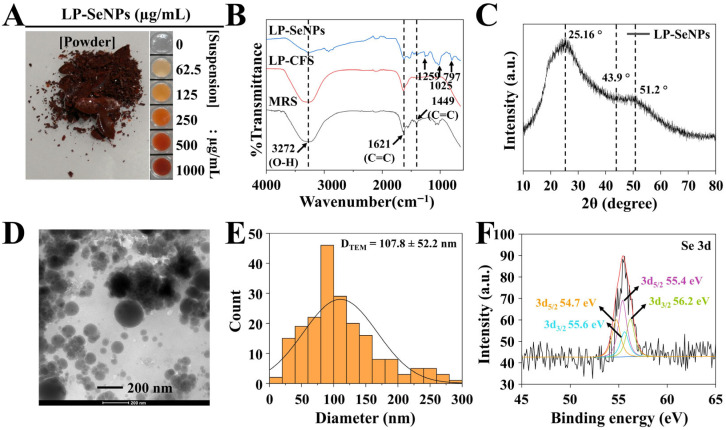
Characterization of LP-SeNPs. (**A**) Representative images of LP-SeNPs in dried powder form and in diluted colloidal dispersion. (**B**) FTIR spectra of LP-SeNPs, LP-CFS, and MRS medium. (**C**) XRD pattern of LP-SeNPs. (**D**) TEM image of LP-SeNPs. (**E**) Particle size distribution analysis derived from TEM images. (**F**) XPS spectrum of LP-SeNPs, with the Se 3d_5_/_2_ and Se 3d_3_/_2_ components indicated by arrows.

Using this CFS-assisted strategy, we successfully achieved extracellular biosynthesis of SeNPs, as confirmed by the characteristic reddish coloration of the dried NPs (Figure 3A), a well-documented optical signature of Se^0^ due to surface plasmon resonance phenomena [41]. The synthesis yielded approximately 7 µg of SeNPs per mL of LP-CFS.

Although several recent studies have reported probiotic- or LAB-mediated SeNPs biosynthesis, significant gaps remain, particularly in the lack of quantitative yield assessments, even among extracellular synthesis approaches [42,43]. Similar limitations are evident in plant extract-based and intracellular biosynthesis methods, where the yield is rarely quantified [44,45]. In this context, our study not only provides one of the few quantitative evaluations of SeNPs production but also represents the first demonstration of extracellular SeNPs biosynthesis using LP-CFS. This positions LP-SeNPs as a novel and robust model system for advancing extracellular, microbially driven NPs synthesis.

### 3.2. Physicochemical Characterization of LP-SeNPs

#### 3.2.1. Characterization of LP-SeNPs

##### FTIR Spectroscopy

The resuspended LP-SeNPs were subjected to FTIR analysis to identify the biomolecular components involved in extracellular synthesis and stabilization. The FTIR spectrum displayed characteristic absorption peaks at 3272, 1621, 1449, 1259, 1025, and 797 cm^−1^ (Figure 3B). Bands at 3272 cm^−1^ (O–H/N–H stretching), 1621 cm^−1^ (amide I, C=O vibration), 1449 cm^−1^ (C–H bending or aromatic skeletal modes), 1259 cm^−1^ (C–N stretching/amide III), and 1025 cm^−1^ (C–O or glycosidic stretching) are indicative of proteins, peptides, and carbohydrate-rich biomolecules typically present in LAB-CFS [46]. The presence of several identical peaks in the spectra of LP-CFS and MRS medium further suggests that proteinaceous and phenolic constituents originating from the culture supernatant directly participate in Se reduction and NP capping.

These observations are consistent with previous reports demonstrated the presence of extracellular proteins in LAB-CFS [47]. Additionally, the absorption band at 797 cm^−1^, assigned to Se–O stretching and bending, likely reflects the formation of an organic coating on the NP surface, potentially derived from carbonyl-containing products generated during ascorbic acid oxidation [41].

Collectively, the FTIR findings strongly support the role of extracellular proteins and associated metabolites in mediating Se(IV)/Se^0^ reduction and stabilizing LP-SeNPs, underscoring the intrinsic capping capability of the LP-CFS matrix.

##### XRD Pattern Analysis

XRD analysis of the LP-SeNPs revealed a broad amorphous halo accompanied by weak Bragg reflections at approximately 25.2°, 43.9°, and 51.2° (Figure 3C). These diffraction positions fall within the 2θ range expected for trigonal or hexagonal Se and correspond well with previously reported patterns for biogenic SeNPs [48]. The pronounced peak broadening confirmed that the NPs were predominantly amorphous, a hallmark of biosynthesized SeNPs stabilized by organic capping layers [49]. Minor reflections within the pattern may further indicate the presence of partially oxidized Se formed under the oxygen-rich culture conditions [50]. Overall, the XRD results highlight the nanoscale and structurally disordered characteristics of the LP-SeNPs, consistent with their biogenic origin.

##### TEM Image Analysis

TEM imaging revealed that LP-SeNPs were predominantly spherical in morphology, consistent with previous reports on biosynthesized SeNPs [51]. However, the NPs were unevenly dispersed and ranged widely in size, from 50 to 200 nm, with an average core diameter of approximately 107 nm (Figure 3D,E). This size heterogeneity likely reflects partial aggregation driven by interactions with the components of the bacterial culture and biomolecules secreted into the extracellular environment [52]. These observations highlight the structural diversity of the LP-SeNPs cores while reinforcing the spherical architecture typical of biogenic SeNPs systems.

##### XPS Analysis

XPS analysis was performed to further elucidate the surface chemical composition of the LP-SeNPs (Figure 3F). The Se 3d region displayed the characteristic Se 3d_5_/_2_ and 3d_3_/_2_ doublet components, separated by ~0.86 eV with a fixed 3:2 area ratio—parameters consistent with established Se reference spectra [53]. Curve fitting required two pairs of Se 3d doublets, each with independently varying full widths at half maximum, indicating the presence of multiple Se with different oxidation states.

Based on reference binding energies, Se^0^ typically appears near 55.1 eV, whereas oxidized species [Se(IV)~59.4 eV and Se(VI)~61 eV] and selenides [Se(II) < 55 eV] exhibit distinct shifts [54]. Notably, Se^0^ can experience slight positive or negative chemical shifts when interacting strongly with organic matrices [55]. The dominant 3d_5_/_2_ peak at 55.4 eV confirmed that the principal species present in LP-SeNPs was Se^0^. Deconvolution of the spectra revealed two distinct Se^0^ states: a lower-energy component near 54.7 eV corresponding to surface Se atoms interacting with the organic capping layer, and a higher-energy component ~55.4 eV attributed to the metallic Se^0^ core.

Together, these XPS findings confirm that LP-SeNPs primarily consist of Se^0^ while exhibiting two chemically distinct Se^0^ environments arising from the core–shell structural organization.

##### Zeta Potential Measurement

Zeta potential measurements were performed to assess the surface charge and colloidal stability of the LP-SeNPs, reflecting the electrostatic potential at the interface between the NP surface and surrounding fluid layer [56]. Typically, NPs exhibiting zeta potentials ≥ ± 30 mV generate sufficient electrostatic repulsion to prevent aggregation, whereas lower absolute values favor particle–particle interactions driven by van der Waals forces, ultimately promoting flocculation [57,58].

LP-SeNPs displayed a zeta potential of −0.12 mV, indicating an extremely low surface charge and, consequently, poor electrostatic stabilization. Such minimal repulsive forces predispose the particles to aggregation, which is consistent with previous observations for biogenic SeNPs synthesized without dedicated stabilizing agents [59]. While this limited electrostatic barrier may facilitate particle clustering and contribute to shifts in hydrodynamic size distribution, it is important to emphasize that endogenous biomolecules present in the LP-CFS, such as peptides, polysaccharides, and diverse metabolites, likely impart partial steric stabilization, thereby mitigating excessive aggregation to some degree.

Nevertheless, these results highlight the need for further optimization to enhance the long-term colloidal stability. Strategies commonly employed in green-synthesized SeNP systems, including polymeric capping (e.g., PEG or PVP), protein-based coatings, and controlled modulation of pH or ionic strength, represent promising avenues for improving NP stability and broadening their potential applications.

#### 3.2.2. Comparison of Physicochemical Properties of LP-SeNPs and Chem-SeNPs

##### UV-Vis Spectroscopy Analysis

**Figure 4:**
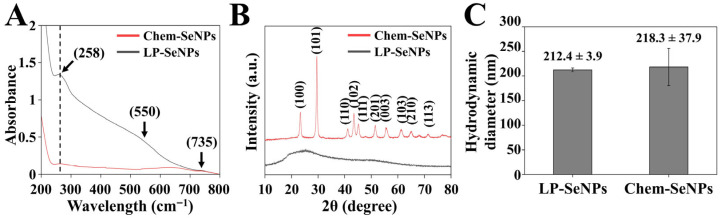
Comparative physicochemical characterization of LP-SeNPs and Chem-SeNPs. (**A**) UV–Vis absorption spectrum of LP-SeNPs and Chem-SeNPs. The dashed line indicates the characteristic absorption peak shared by both types of SeNPs. (**B**) XRD patterns of LP-SeNPs and Chem- SeNPs. (**C**) Hydrodynamic diameter of LP-SeNPs and Chem-SeNPs. The diameter was measured by DLS.

The UV-Vis spectrum of the LP-SeNPs exhibited a pronounced absorption peak at 258 nm within the 200–800 nm range (Figure 4A), a signature feature consistent with SeNPs synthesized via ascorbic acid reduction [24]. Notably, additional shoulders at approximately 550 and 735 nm were detected, which are characteristic of NPs aggregation. This spectral profile corroborates earlier findings that link the SeNPs size distribution and colloidal stability of SeNPs to distinct UV–Vis absorption behaviors [60]. Collectively, these features indicate that LP-SeNPs possess a heterogeneous morphology dominated by well-dispersed NPs, whereas a smaller subset of aggregated particles contributes to the higher-wavelength shoulders. In contrast, Chem-SeNPs displayed only a weak absorption band near 250 nm, suggesting a markedly lower abundance of Se^0^. The complete absence of aggregation-associated shoulders further underscores the distinct physicochemical nature of Chem-SeNPs, highlighting substantial differences in particle formation, stability, and overall quality compared to LP-SeNPs.

##### XRD Analysis

XRD analysis comparing the crystalline structures of the LP-SeNPs and Chem-SeNPs (Figure 4B) demonstrated a striking distinction between the two SeNPs types. Chem-SeNPs displayed well-defined, sharp Bragg reflections corresponding to the (100), (101), (110), (102), (111), (201), (003), (103), (210), and (113) planes, which is consistent with the Joint Committee on Powder Diffraction Standards (JCPDS) card No. 06-0362 [61], thereby confirming their highly crystalline nature. In contrast, the LP-SeNPs exhibited only broad, low-intensity peaks, indicative of limited long-range order. Although both samples shared similar diffraction peak positions, the markedly broader pattern of the LP-SeNPs underscores their predominantly amorphous structure. This difference in peak sharpness and intensity highlights fundamental disparities in the structural organization and crystallinity of LP-SeNPs and Chem-SeNPs.

##### DLS Analysis

DLS analysis further elucidated the pronounced differences in the hydrodynamic size distribution of LP-SeNPs and Chem-SeNPs (Figure 4C). LP-SeNPs exhibited a mean hydrodynamic diameter of 212.4 ± 3.9 nm, indicating a highly uniform particle population under diluted conditions. The discrepancy observed between the DLS and TEM results (Figure 3D,E) for LP-SeNPs is consistent with the distinct principles of the two techniques. While TEM captures the physical core size, DLS measures the hydrodynamic diameter, which incorporates the surrounding solvation shell and the biomolecular capping layer characteristic of biogenic NPs [62]. These findings indicate that despite the structural heterogeneity at the core level, LP-SeNPs display remarkably consistent hydrodynamic behavior in suspension. In contrast, Chem-SeNPs displayed a substantially broader and more heterogeneous size distribution, with a mean diameter of 218.3 ± 37.9 nm, reflecting greater viability in particle size and stability. These findings demonstrated that LP-SeNPs possessed a slightly smaller and significantly more homogeneous hydrodynamic profile than Chem-SeNPs, underscoring the stabilizing influence of biomolecular capping inherent to the biogenic synthesis route.

From all above physicochemical analyses, we demonstrated that the successful biosynthesis of LP-SeNPs, which display the hallmark optical, structural, and morphological attributes of biogenic SeNPs. The LP-SeNPs exhibited distinct biomolecular signatures arising from protein- and phenolic-based capping agents, an amorphous structural profile, and a uniformly spherical nanoscale morphology. Although their relatively low zeta potential suggests limited intrinsic colloidal stability, the LP-SeNPs maintained consistent hydrodynamic behavior under diluted conditions. In a direct comparison, LP-SeNPs differed markedly from Chem-SeNPs across multiple physicochemical dimensions, including substantially higher UV–Vis absorbance intensity, broader and less crystalline XRD patterns, and a more uniform hydrodynamic size distribution. These pronounced distinctions highlight the fundamental impact of biogenic synthesis on NPs nucleation, growth, and stabilization, ultimately producing LP-SeNPs with unique and biologically driven structural characteristics.

#### 3.2.3. Time-Dependent DLS Analysis

**Figure 5:**
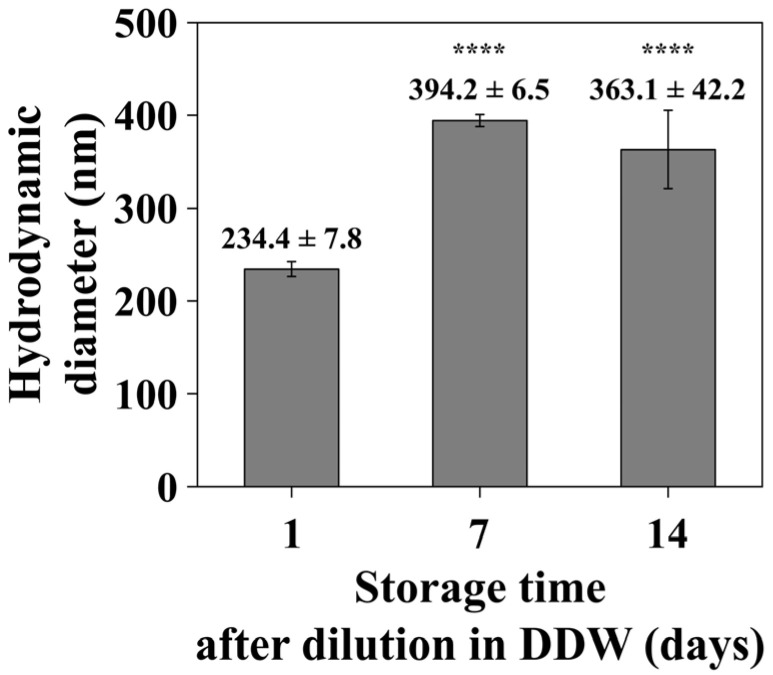
Time-dependent hydrodynamic diameter of LP-SeNPs. The DLS measurements were conducted on LP-SeNPs samples stored for 1, 7, and 14 days following dilution with DDW to monitor changes in hydrodynamic diameter and evaluate the stability of the LP-SeNPs over time. Statistical significance: **** *p* < 0.0001.

Although all LP-SeNPs characterizations in this study were performed immediately after resuspending the powdered material in DDW, it was important to evaluate their reproducibility and storage-related stability after dilution. To this end, time-dependent DLS measurements were conducted on samples stored for 1, 7, and 14 days in DDW (Figure 5). The 1-day sample showed a hydrodynamic diameter of 234.4 ± 7.8 nm, consistent with the size of freshly prepared LP-SeNPs (Figure 4C). However, the 7- and 14-day samples exhibited an approximately 1.5-fold increase in hydrodynamic diameter, indicating progressive NPs aggregation and a gradual decline in colloidal stability over time.

This trend aligns with previous reports that biosynthesized NPs often bear biomolecular surface coatings susceptible to structural or chemical changes during storage, promoting aggregation and reducing the long-term stability [63]. Collectively, the observed increase in particle size suggests that the current dilution and storage conditions are suboptimal and require optimization to improve the long-term stability of LP-SeNPs.

### 3.3. Antibacterial Potential of LP-SeNPs

#### 3.3.1. Evaluation of Antibacterial Activity of LP-SeNPs 

The antibacterial activities of Chem-SeNPs and LP-SeNPs were evaluated by determining their MICs using a microdilution assay (Figure S1). Both Gram-negative (*A. baumannii*, *E. coli*, *K. pneumoniae*, and *P. aeruginosa*) and Gram-positive strains (MRSA, *S. aureus* ATCC 25923, *S. epidermidisa*, and *S. saprophyticus*) strains were examined across a concentration range of 0–100 µg/mL (Table 1). Chem-SeNPs showed no antibacterial activity against any of the tested bacterial strains. Similarly, all Gram-negative strains retained substantial growth in the presence of LP-SeNPs even at the highest tested concentration (100 µg/mL), indicating negligible or no activity. In contrast, *S. aureus* displayed measurable sensitivity, with an MIC of 50 µg/mL.

**Figure 6:**
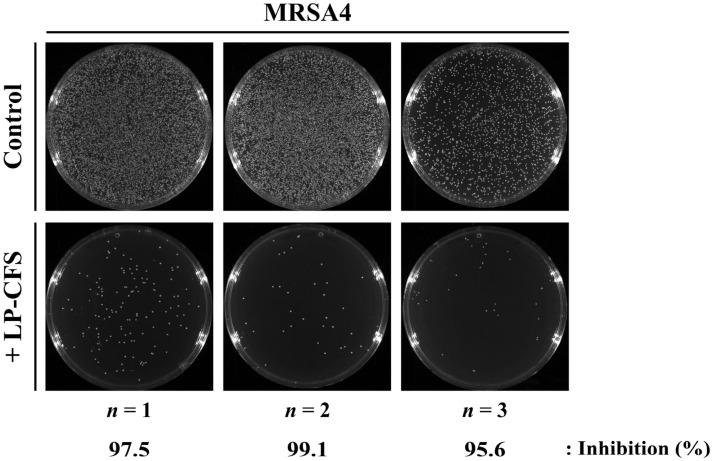
Antibacterial activity of LP-CFS against MRSA4. CFU plate images for samples treated with or without LP-CFS at a 10^−5^ dilution, along with the corresponding growth inhibition efficiency, are shown. The inhibition (%) was calculated as the number of colonies in +LP-CFS plate divided by those of the untreated control. Individual replicates (*n* = 3) are displayed. Images were captured using a ChemiDoc^TM^ MP system (Bio-Rad) and analyzed with Image Lab^TM^ software (Bio-Rad).

Given the previously reported antibacterial activity of LP-CFS against MRSA [64], we first assessed the effect of LP-CFS alone on MRSA4, a representative clinical MRSA isolate. The cells were incubated with or without LP-CFS for 3 h and then subjected to CFU enumeration (Figure 6). In the 10^−5^ dilution plates, LP-CFS treatment resulted in a marked reduction in viable cell counts. The growth inhibition efficiency was 97.44 ± 1.43%. These findings confirm that LP-CFS possesses intrinsic antibacterial activity against MRSA strains, which may contribute to the antibacterial properties of LP-SeNPs.

Following the confirmation of LP-CFS antimicrobial activity, the antibacterial efficacy of LP-SeNPs was evaluated against six MRSA isolates. LP-SeNPs displayed potent activity, with MIC values as low as 0.5 µg/mL—approximately 100-fold lower than those observed for *S. aureus*. Other Gram-positive species, including *S. epidermidis* and *S. saprophyticus*, showed higher MICs of 50 µg/mL and 8 µg/mL, respectively. Compared with previously reported LAB-synthesized SeNPs—which often showed limited antibacterial characterization, broad-spectrum but relatively high MIC values, or relied on intracellular synthesis [42,43,47,65,66,67]. Following confirmation of LP-CFS antimicrobial activity, the antibacterial efficacy of LP-SeNPs was evaluated against six MRSA isolates. LP-SeNPs displayed potent activity, with MIC values as low as 0.5 µg/mL—approximately 100-fold lower than those observed for *S. aureus*. Other Gram-positive species, including *S. epidermidis* and *S. saprophyticus*, showed higher MICs of 50 µg/mL and 8 µg/mL, respectively.

To determine whether LP-CFS alone could enhance the activity of chemically synthesized SeNPs, Chem-SeNPs (1 mg/mL) were mixed with 1 mL of LP-CFS (Section 2.2) and tested for antibacterial activity. The LP-CFS–mixed Chem-SeNPs showed no activity against *S. aureus* or MRSA (Figure S2), yielding responses identical to those of Chem-SeNPs alone (Table 1). These findings demonstrated that the potent antibacterial activity of LP-SeNPs cannot be attributed to residual LP-CFS components; instead, they arise from specific biomolecular interactions formed during the biosynthesis process.

Based on previous studies, the pronounced Gram-positive selectivity of LP-SeNPs can be attributed to several interconnected factors. Structurally, Gram-positive bacteria possess a thick peptidoglycan layer and lack an outer lipopolysaccharide membrane, allowing more direct interactions between the cell surface and NPs [68]. In addition, NP features such as size, morphology, and surface chemistry critically influence the antibacterial performance [69,70]. The biogenic nature of LP-SeNPs further enhances this selectivity, as LP produces antibacterial metabolites and its CFS supplies bioactive components that associate with the NP surface. Consistent with the above aspects, FTIR and XPS analyses (Figure 3) confirmed the presence of an LP-CFS-derived organic capping layer, which likely strengthened the NPs–cell surface interactions and contributed to the superior antimicrobial activity of LP-SeNPs.

In summary, the potent and highly selective antibacterial activity of LP-SeNPs arises from the combined effects of their favorable physicochemical properties, the biogenic synthesis route, and LP-CFS–derived organic capping layer. These factors work synergistically to enhance interactions with Gram-positive bacterial surfaces, resulting in markedly improved efficacy compared to chemically synthesized counterparts. The strong activity against MRSA, together with limited effects on Gram-negative species, highlights LP-SeNPs as promising targeted, eco-friendly antimicrobial agents with the potential to preserve the host microbiota and reduce the risk of antimicrobial resistance [71,72].

#### 3.3.2. Determination of Bactericidal Activity Using Time–Kill Curve Assays

**Figure 7:**
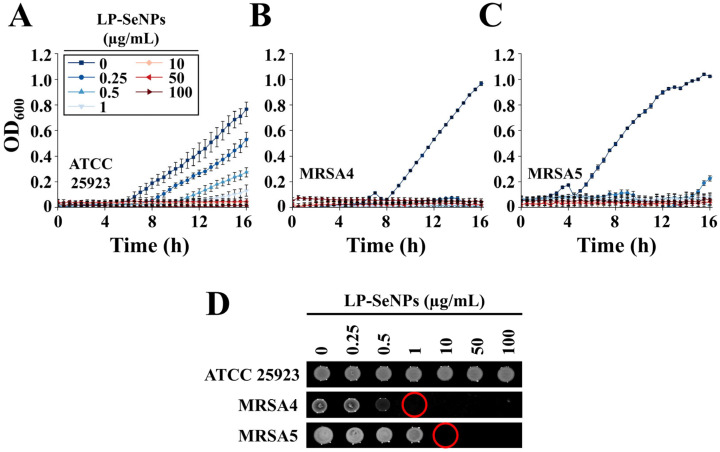
Antibacterial activity of LP-SeNPs. (**A**–**C**) Time-kill assays of LP-SeNPs against (**A**) *S. aureus* (ATCC 25923), (**B**) MRSA4, and (**C**) MRSA5. Bacterial suspensions were treated with LP-SeNPs at varying concentrations, and 16 h growth was monitored by OD_600_ measurement. (**D**) Viability of LP-SeNPs-treated *S. aureus* (ATCC 25923), MRSA4, and MRSA5. Endpoint viability from the growth (**C**) was assessed by spot plating on LB agar. Red circles indicate the bactericidal concentration of LP-SeNPs against MRSA4 and MRSA5, while no bactericidal activity was observed for S. *aureus*. A representative dataset from triplicate experiments is shown.

The bactericidal activity of LP-SeNPs against *S. aureus* (ATCC 25923) and MRSA strains was assessed using time-kill curve assays (Figure 7A–C and Figure S3). *S. aureus*, MRSA4, and MRSA5 cultures were exposed to LP-SeNPs (0–100 μg/mL) for 16 h under shaking conditions, and viability was quantified by spot plating on LB agar. MRSA4 and MRSA5 were selected due to their low MIC values (0.5 μg/mL; Table 2) and documented resistance to penicillin-class antibiotics [23].

LP-SeNPs displayed marked differences in bactericidal thresholds between the non-resistant and MRSA strains. For *S. aureus*, the concentration required to achieve a 90% reduction in viable cells (MBC_90_) exceeded 10 μg/mL. In contrast, MRSA4 and MRSA5 exhibited much lower MBC_90_ values of 0.25 μg/mL and 0.5 μg/mL, respectively (Figure 7A–C). Viability assays confirmed that these concentrations represented the true bactericidal endpoints, as no colonies were detected at MBC_90_ (Figure 7D).

Although LP-SeNPs primarily showed bacteriostatic effects under shaking conditions, their pronounced and selective bactericidal activity against MRSA strains was evident, demonstrating nearly 100-fold greater potency than against the non-resistant strain. This high degree of selectivity highlights the strong therapeutic potential of LP-SeNPs as targeted antimicrobial agents against MRSA.

### 3.4. Cytotoxicity of LP-SeNPs

**Figure 8:**
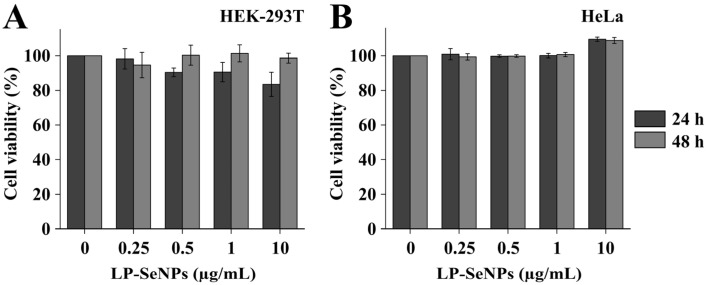
Cytotoxicity of LP-SeNPs. Cytotoxicity of LP-SeNPs toward (**A**) HEK-293T cells and (**B**) HeLa cells (0–10 µg/mL) after 24 h and 48 h, measured by MTT assay.

The cytotoxicity of LP-SeNPs was evaluated using the MTT assays in HEK-293T and HeLa cells (Figure 8). HEK-293T cells were selected as a standard model for human kidney-derived cells commonly used in NP toxicity studies [73], whereas HeLa cells were included because of their widespread use in cytotoxicity assessments, including evaluations of SeNPs [74]. LP-SeNPs were tested across a broad concentration range, from 0 to 20× the MRSA MIC (10 µg/mL), and cell viability was monitored over 2 days to assess both concentration- and time-dependent responses.

The LP-SeNPs exhibited minimal cytotoxicity in both cell lines, even at the highest concentrations tested. In HEK-293T cells, only the maximum dose produced a modest ~25% reduction in viability, whereas MIC-level concentrations had negligible effects (Figure 8A). In HeLa cells, LP-SeNPs showed minimal cytotoxicity at all the tested concentrations (Figure 8B). Consistent with these observations, the calculated IC_50_ values for both cell lines were substantially higher than the bactericidal concentrations required to inhibit MRSA (IC_50_ > 10 µg/mL), indicating a wide therapeutic margin. The low cytotoxicity and high IC_50_ values underscore the selective antibacterial action of LP-SeNPs, which likely reflects fundamental differences in membrane composition, cell envelope structure, and nanoparticle internalization pathways between prokaryotic and eukaryotic cells [75,76,77]. Collectively, these findings support the biocompatibility of LP-SeNPs and highlight their potential as safe and effective antibacterial agents for therapeutic applications.

### 3.5. Assessment of the Role of LP-SeNPs–Associated Biomolecules in Antibacterial Activity

Biosynthesis of NPs enables the transfer of bioactive properties from biological source materials to the resulting NPs [78]. In particular, CFS-mediated NP synthesis often enriches proteins and nucleic acids from CFS, allowing these biomolecules to associate with synthesized NPs [79,80]. To assess whether the antibacterial activity of LP-SeNPs is influenced by such LP-derived biomolecules, we performed MIC assays on LP-SeNPs against MRSA4, a representative MRSA strain, with or without pre-treatment with proteinase K or DNase, which selectively remove protein- or DNA-associated components, respectively.

**Figure 9:**
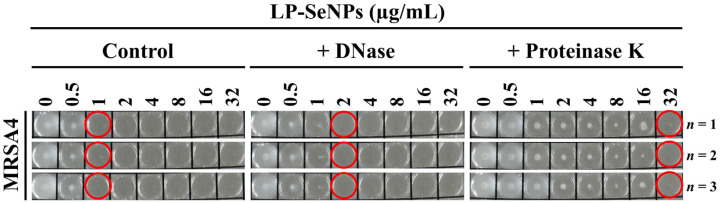
Effect of DNA- and protein-associated biomolecules on the activity of LP-SeNPs. Antibacterial activity against MRSA4 was assessed using the MIC assay with or without DNase or proteinase K treatment, as described in *Materials and Methods*. *n* indicates the number of experiments. Red circles indicate the MIC values of LP-SeNPs for each treatment condition.

As shown in Figure 9, DNase-treated LP-SeNPs showed a two-fold increase in the MIC, whereas proteinase K–treated LP-SeNPs displayed a 32-fold increase relative to the control. These results indicate that protein-associated components are the major functional contributors to the antibacterial activity of LP-SeNPs.

The substantial loss of activity following protein degradation suggests that surface-associated proteins are critical for mediating interactions with bacterial cells, likely by facilitating cell wall and membrane disruption, and enhancing ROS-mediated stress. In contrast, the modest MIC increase after DNase treatment implies that nucleic acids play a secondary role, potentially contributing to NP stability or surface charge, thereby indirectly supporting antibacterial function. These findings are consistent with previous reports showing that biomolecules retained on biosynthesized NPs can enhance their biological activities. Overall, these results highlight the importance of preserving surface-bound proteins during LP-SeNPs biosynthesis to maximize antibacterial efficacy and may guide the development of next-generation biogenic SeNPs with optimized functional coatings. However, the specific proteins and nucleic acid species responsible remain unidentified, and further molecular-level studies are needed to elucidate their precise contributions to LP-SeNPs antibacterial mechanisms.

### 3.6. Antibacterial Mechanisms of LP-SeNPs Against MRSA

#### 3.6.1. Synergistic Antibiotic Screening

The four major antibacterial pathways include inhibition of protein synthesis, inhibition of cell wall synthesis, disruption of cell membrane integrity, and inhibition of nucleic acid synthesis [81]. To investigate which of these pathways are influenced by LP-SeNPs, representative antibiotics targeting each mechanism were selected to assess potential synergistic effects against six MRSA isolates: COL (membrane disruption), ERY/TET (protein synthesis inhibition via the 50 S and 30 S ribosomal subunits), IMP (cell wall synthesis inhibition), and RIF (nucleic acid synthesis inhibition). The MIC of these antibiotics against each isolate were determined (Figure S4 and Table 2).

**Figure 10:**
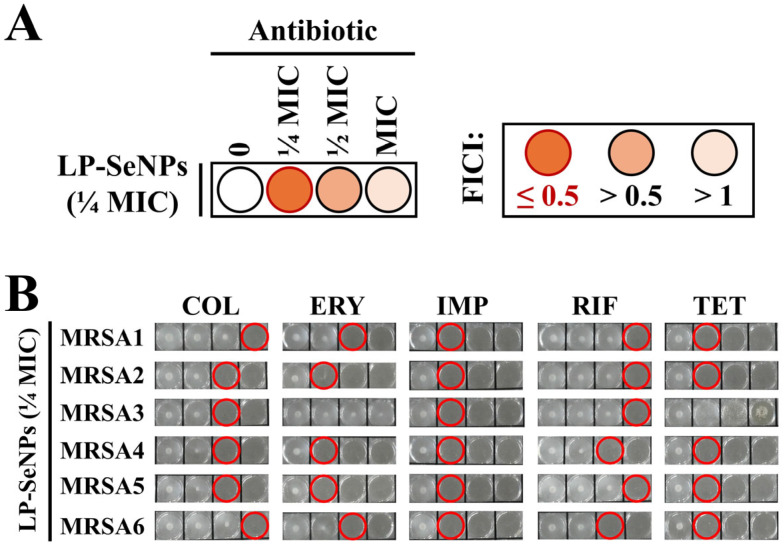
Identification of synergistic antibiotics to LP-SeNPs. (**A**) Schematic representation of FICI interpretation. Red circles indicate FICI ≤ 0.5, denoting synergistic interactions. (**B**) Representative results of LP-SeNPs combined with selected antibiotics against six MRSA isolates. Red circles indicate MIC values of individual antibiotics in combination treatments. COL, colistin; ERY, erythromycin; IMP, imipenem; RIF, rifampicin; TET, tetracycline.

A schematic overview of the FICI-based plate assay is shown in Figure 10A. Synergy between LP-SeNPs and the tested antibiotics was evaluated in triplicate, with the representative outcomes shown in Figure 10B and the full data presented in Figure S5. Synergistic interactions were defined using the FICI, where FICI ≤ 0.5 (corresponding to a ≥4-fold MIC reduction in the presence of ¼ MIC LP-SeNPs) and FICI > 0.5 indicated synergistic and no synergistic effect, respectively. The resulting FICI values are listed in Table 3.

As shown in Table 3, LP-SeNPs markedly potentiated the antibacterial activity of IMP across all six MRSA isolates, yielding a uniform 4-fold reduction in MIC values. Because IMP inhibits cell wall synthesis by disrupting peptidoglycan cross-linking [82], this robust and consistent synergistic effect strongly indicates that LP-SeNPs compromise cell wall integrity, thereby facilitating antibiotic entry. This interpretation is supported by previous studies reporting SeNPs-induced surface deformation and increased cell envelop permeability in Gram-positive bacteria, which enhance the performance of β-lactam antibiotics [83]. Taken together, these findings suggest that interference with the bacterial cell wall as the primary antimicrobial mechanism of LP-SeNPs.

Synergistic interactions were also noted with the protein synthesis inhibitors ERY and TET, which target the 50 S and 30 S ribosomal subunits, respectively [84,85]. Unlike IMP, these effects were more variable and strain-dependent: ERY showed synergy with MRSA2, MRSA4, and MRSA5, whereas TET enhanced the activity against all isolates except MRSA3 (Table 3). This heterogeneity suggests that LP-SeNPs internalization and subsequent intracellular interactions may differ among strains, potentially reflecting variations in the membrane composition, surface charge, or efflux capacity. Thus, synergy with protein synthesis inhibitors may represent a secondary mechanism that becomes relevant only when LP-SeNPs traverse the cytoplasmic membrane and interact with ribosomes or protein-folding pathways.

In contrast, LP-SeNPs showed no meaningful synergy with COL or RIF, as the MIC changes did not exceed a two-fold reduction (Table 3). The absence of synergy with COL indicates that LP-SeNPs do not primarily act through membrane depolarization or disruption of lipid bilayers, whereas the lack of synergy with RIF suggests that nucleic acid synthesis is not a major target.

Collectively, these results demonstrate that LP-SeNPs selectively amplify the activity of antibiotics acting on cell membrane synthesis and, to a lesser extent, those inhibiting protein synthesis. The consistent synergy with IMP, together with the morphological evidence of envelope disruption, provides compelling support that LP-SeNPs exert their principal antibacterial effect by damaging the bacterial cell wall and membrane. Secondly, strain-specific interactions with intracellular targets may further contribute under certain conditions, offering a multifaceted antibacterial mode of action that complements conventional therapeutics.

#### 3.6.2. Morphological Characterization of MRSA Cells Treated with LP-SeNPs

**Figure 11:**
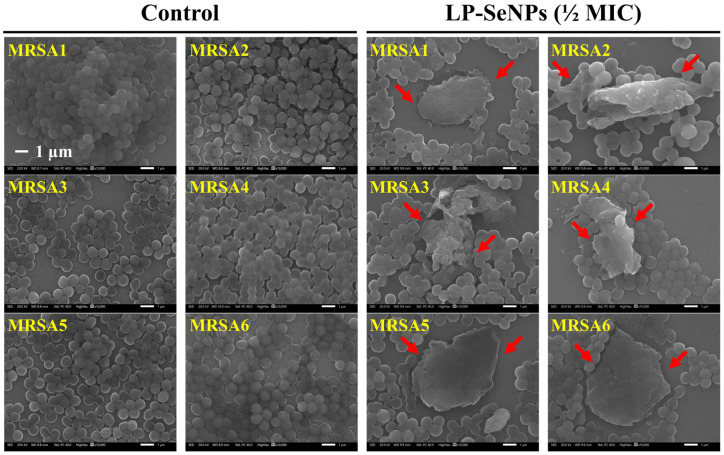
SEM images of six MRSA isolates treated with or without ½ MIC LP-SeNPs for 4 h. Red arrowheads indicate the disrupted bacterial cell membrane.

Biosynthesized SeNPs are known to exert antibacterial activity primarily through disruption of the bacterial cell wall and membrane, ultimately causing the leakage of intracellular components and cell death [83]. In line with this established mechanism, SEM image analysis of MRSA cells exposed to ½ MIC of LP-SeNPs for 3 h (Figure 11) revealed clear evidence of membrane disruption in all six isolates.

Untreated MRSA cells exhibited the typical spherical morphology of healthy *S. aureus*, characterized by smooth, intact surfaces and clearly defined cell boundaries, consistent with previous descriptions of normal *Staphylococcal* architecture [86]. In contrast, the LP-SeNP-treated cells showed extensive morphological deterioration, including distorted or shrunken shapes, surface ruptures, pronounced pitting, and partial collapse or tearing of the cell envelope. In several isolates, deep surface depressions and visible leakage of cytoplasmic material were evident, indicating a severe compromise of membrane integrity and progressive structural breakdown.

These observations provide strong visual confirmation that LP-SeNPs inflict direct physical damage to the MRSA cell envelope, supporting their proposed mode of action and correlating with the synergistic enhancement observed with cell-wall–targeting antibiotics. Furthermore, the pronounced envelope disruption observed in MRSA closely aligns with the strong synergistic activity of LP-SeNPs and IMP, a β-lactam antibiotic that inhibits peptidoglycan cross-linking. This correspondence reinforces the conclusion that LP-SeNPs primarily compromise cell wall integrity, likely through interactions with negatively charged surface components or by inducing oxidative stress that destabilizes the peptidoglycan matrix.

Notably, *S. aureus* (ATCC 25923) exposed to the same LP-SeNPs concentration retained intact morphology with only minor surface alterations (Figure S6). This contrast indicates that the membrane-disruptive effects of LP-SeNPs—and the resulting antibiotic synergy—are substantially more pronounced in MRSA, suggesting a heightened vulnerability associated with resistance-associated changes in the cell envelope.

Overall, these findings demonstrate that LP-SeNPs exert their dominant antibacterial effects of MRSA through targeted disruption of the cell wall and membrane. The selective structural damage in MRSA, combined with the enhanced susceptibility to IMP, highlights the potential of LP-SeNPs as a complementary antimicrobial strategy that potentiates β-lactam efficacy by increasing cell wall permeability and weakening envelope integrity.

#### 3.6.3. Characterization of Total Cell Proteins in MRSA Treated with LP-SeNPs

**Figure 12:**
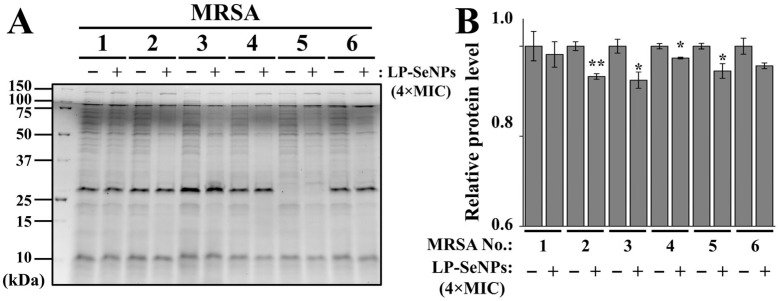
Effect of LP-SeNPs on total cellular protein levels. (**A**) Total cellular protein profiles of six MRSA isolates treated with or without 4-fold MIC LP-SeNPs, separated on 12% TGX stain-free gels (Bio-Rad) and visualized. One representative dataset from triplicate experiments is shown. (**B**) Relative protein levels quantified using Image Lab^TM^ Software (ver. 5.2.1; Bio-Rad). Statistical significance: * *p* < 0.05, ** *p* < 0.01.

To evaluate whether LP-SeNPs influenced protein synthesis, total cellular proteins from six MRSA isolates treated with 4× MIC LP-SeNPs (2–4 µg/mL) were analyzed by SDS-PAGE (Figure 12A). All the samples were normalized to equal biomass prior to loading to ensure an accurate comparison. Densitometric analysis of lane intensities was subsequently performed to quantify potential treatment-related differences in overall protein content (Figure 12B).

Both protein gel imaging and quantitative intensity analysis revealed minimal differences between the LP-SeNPs–treated and untreated groups, with the relative protein level ratios ranging from 0.02 to 0.08. Although several MRSA strains showed statistically significant reductions following LP-SeNPs exposure (* *p* < 0.05), these changes were small in magnitude, indicating that LP-SeNPs did not substantially alter the overall protein expression under the conditions tested.

The inconsistent synergistic effects observed with erythromycin and tetracycline across the six MRSA isolates indicate that interference with protein synthesis is not the primary antibacterial mechanism of LP-SeNPs. Because protein synthesis inhibition requires intracellular access [87], whereas cell wall disruption is initiated extracellularly the collective evidence supports the conclusion that LP-SeNPs act predominantly by compromising the cell wall, with any effects on protein synthesis likely reflecting the secondary or indirect consequences of membrane permeabilization.

Nonetheless, these mechanistic insights are preliminary, as protein-related analyses were performed at a single high concentration (4× MIC) using conventional SDS-PAGE, a technique that is insufficient for detecting subtle, transient, or low-abundance alterations. Comprehensive elucidation of the molecular targets of LP-SeNPs will require high-resolution, quantitative proteomic approaches across multiple concentrations and time points to capture the full spectrum of cellular responses underlying their anti-MRSA activity.

#### 3.6.4. ROS Production Ability of LP-SeNPs

Numerous studies have reported that biogenic SeNPs exert antibacterial activity through the induction of ROS [69,83], with NP-mediated ROS generation being recognized as a major contributor to their antimicrobial efficacy [88]. To assess the ROS-producing capability of LP-SeNPs, we performed a DCFH-DA assay using *S. aureus* (ATCC 25923), MRSA4, and MRSA5. Bacterial cultures were treated with increasing concentrations of LP-SeNPs (0–50 μg/mL) or Chem-SeNPs (100 μg/mL) in the presence of 5 mM DCFH-DA for 3 h. MRSA4 and MRSA5 were selected based on their relatively low MIC values (Table 1) and documented resistance to penicillin-class antibiotics [23].

**Figure 13:**
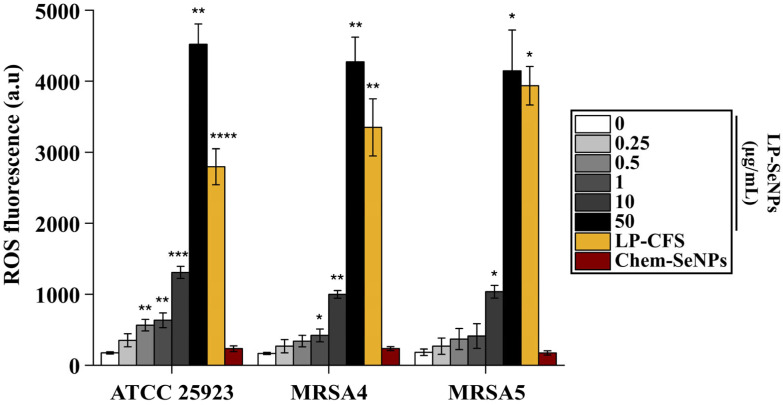
ROS generation by LP-SeNPs, LP-CFS and Chem-SeNPs. ROS levels in *S. aureus* (ATCC 25923), MRSA4, and MRSA5 were evaluated using bacterial suspensions treated with LP-SeNPs (0–50 µg/mL), LP-CFS (100 µL), or Chem-SeNPs (100 µg/mL) in the presence of 5 µM DCFH-DA. Fluorescence intensity (Ex/Em = 485/520 nm) was measured and normalized to untreated controls. Statistical significance: * *p* < 0.05, ** *p* < 0.01, *** *p* < 0.001, **** *p* < 0.0001.

As shown in Figure 13, all three strains exhibited a clear, concentration-dependent increase in ROS production following LP-SeNPs treatment, with fluorescence intensities reaching ~4500-fold above untreated controls at 50 μg/mL. However, the extent of ROS generation did not correspond to the MIC values of the strains (Table 1), indicating that ROS induction alone does not determine antibacterial susceptibility.

Because LP-CFS serves as a biological source for LP-SeNPs synthesis, we next examined its contribution to ROS generation. ROS production induced by LP-CFS alone, applied at 100 μL, equivalent to ~1.5% of the CFS content present in 50 μg/mL LP-SeNPs, was substantial across all strains and was comparable to the levels generated by 10–50 μg/mL LP-SeNPs, with only minor strain-specific differences (Figure 13). In contrast, Chem-SeNPs produced ROS at levels similar to LP-SeNPs only at a markedly lower concentration (0.25 μg/mL), suggesting that the selenium core exhibits limited intrinsic ROS-generating ability. Collectively, these results indicate that the elevated ROS levels associated with LP-SeNPs treatment are largely attributable to CFS-derived biomolecules rather than the SeNPs core itself.

When integrated with the SEM observations and protein expression analyses (Figure 11 and Figure 12), these findings support a mechanistic model in which LP-SeNPs exert their primary antibacterial effect through disruption of the cell wall and membrane. ROS generation appears to act as a secondary, synergistic contributor, enhancing but not solely responsible for the antibacterial outcome. This combined action of physical envelope damage and oxidative stress aligns with the multimodal activity reported for other biogenic SeNPs [69].

## 4. Conclusions

In conclusion, this study demonstrated the successful extracellular biosynthesis of SeNPs using LP-CFS, yielding LP-SeNPs with uniform size, stable morphology, and functional biomolecular coatings. As summarized in Table 4, this LP-CFS-based approach offers clear advantages over conventional intracellular synthesis, including the elimination of cell lysis and toxic reagents, simplified NPs recovery, and eco-friendly, pharmaceutically relevant production.

Mechanistic investigations clarified both the origin and nature of the antibacterial activity of LP-SeNPs. Although LP-CFS alone possessed intrinsic antibacterial and ROS-inducing properties, its addition to Chem-SeNPs did not enhance antimicrobial activity, confirming that the potent effects of LP-SeNPs arose from synergistic interactions between the selenium core and CFS-derived biomolecules formed specifically during biosynthesis. LP-SeNPs selectively targeted Gram-positive bacteria—including multiple MRSA isolates—with MICs as low as 0.5 µg/mL, while Gram-negative strains remained largely unresponsive.

Antibiotic synergy testing and SEM analysis identified the disruption of the bacterial cell wall as the primary antibacterial mechanism of LP-SeNPs. Secondary or synergistic contributions have been observed for biomolecular capping effects, protein synthesis interference, and ROS generation. Consistent with this selective mechanism, LP-SeNPs showed minimal cytotoxicity toward mammalian cells (IC_50_ > 10 µg/mL), including the HEK-293T and HeLa cell lines.

Time-dependent DLS analysis further revealed progressive aggregation following dilution and a low zeta potential, indicating limitations in colloidal stability that may affect long-term reproducibility. Future formulation strategies are critical to address this challenge.

Overall, this study establishes LP-SeNPs as a sustainable, biocompatible, and highly potent antibacterial platform. Their primary mode of action involves targeted disruption of the Gram-positive cell envelope, with biomolecular capping enhancing both efficacy and selectivity. These findings warrant further investigation of in vivo activity, pharmacokinetics, antibiotic synergy, and stability optimization to advance LP-SeNPs toward therapeutic development against MDR MRSAs.

## Figures and Tables

**Table 1:** MICs of Chem-SeNPs and LP-SeNPs against bacterial strains.

Species	^1^ Strain Identification Number	MIC (µg/mL)	
		Chem- SeNPs	LP- SeNPs
*Escherichia coli*	ATCC 25922	>100	>100
*Klebsiella pneumoniae*	NCCP 16285	>100	>100
*Acinetobacter baumannii*	ATCC 19606	>100	>100
*Pseudomonas aeruginosa*	ATCC 27853	>100	>100
*Staphylococcus aureus*	ATCC 25923	>100	50
^2^ Clinical isolates of methicillin-resistant *S. aureus*	MRSA1	>100	0.5
	MRSA2	>100	1
	MRSA3	>100	1
	MRSA4	>100	0.5
	MRSA5	>100	0.5
	MRSA6	>100	1
*Staphylococcus epidermidis*	ATCC 14990	>100	50
*Staphylococcus saprophyticus*	ATCC 15305	>100	8

^1^ ATCC and NCCP represent American Type Culture Collection (https://www.atcc.org/) and National Culture Collection for Pathogens (https://nccp.kdca.go.kr/), respectively. ^2^ The Strain information is provided in a previous report [23].

**Table 2:** MIC results of variable antibiotics against MRSA isolates.

Antibiotic	Mechanism of Action	Cellular Target	MIC (µg/mL) Against MRSA Strains (-LP-SeNPs)					
			1	2	3	4	5	6
COL	Cell membrane disruption	LPS binding	64	64	32	64	64	32
ERY	Protein synthesis inhibition	50 S ribosome binding	1	1	>100	2	>100	1
IMP	Cell wall synthesis inhibition	PBP binding	0.5	0.5	0.5	0.5	0.5	0.5
RIF	Nucleic acid synthesis inhibition	DNA dependent RNA polymerase target	0.25	0.25	0.25	0.25	0.25	0.25
TET	Protein synthesis inhibition	30 S ribosome binding	1	0.5	>100	0.5	1	2

**Table 3:** Synergy test results of ¼ MIC LP-SeNPs treated variable antibiotics against MRSA isolates.

Antibiotic	MIC Against MRSA Strains (+LP-SeNPs) (µg/mL)						Synergy Phenotype
	1	2	3	4	5	6	
COL	64	2	16	32	32	32	No synergy
ERY	0.5	0.25	>100	0.5	25	0.5	Isolates dependent synergy
IMP	0.13	0.13	0.13	0.13	0.13	0.13	Synergy
RIF	0.25	0.25	0.25	0.25	0.25	0.13	No synergy
TET	0.25	0.13	>100	0.13	0.25	0.5	Isolates dependent synergy

**Table 4:** Comparative overview of intracellular and LP-CFS-based SeNPs (LP-SeNPs).

Parameter	Intracellular Synthesis [22]	LP-SeNPs (This Study)
Synthesis strategy	Intracellular biotransformation within bacterial cells	Extracellular, CFS-mediated biogenic reduction
Production mechanism	Enzyme-mediated intracellular reduction during growth	Enzyme- and peptide-mediated extracellular reduction in CFS [21]
Cell Lysis reagents (Enzyme or chemicals)	Requires lysozyme, SDS, or 1-octanol	Not required
Purification steps	Multi-steps: cell lysis, centrifugation and solvent extraction	Single step: centrifugation
Localization of SeNPs	Intracellular; associated with cytoplasmic debris	Extracellular, freely suspended in CFS
Yield	Not quantified; low recovery	~7 µg/mL of CFS
Antibacterial activity	Low	High
Target species	*S. aureus* and *E. coli* (non-specific)	MRSA (specific)
Mechanistic insight	Not characterized	Cell wall disruption (Major)

## Data Availability

Data is contained within the article or Supplementary Materials.

## Supplementary Materials

The following supporting information can be downloaded at https://www.mdpi.com/article/10.3390/pharmaceutics18010014/s1, Figure S1. Images of the MIC assay for Chem-SeNPs and LP-SeNPs, Figure S2. Images of the MIC assay for Chem-SeNPs co-treated with LP-CFS, Figure S3. Antibacterial activity of LP-SeNPs, Figure S4. Images of the MIC assay for various antibiotics, Figure S5. Synergistic effects of LP-SeNPs (¼ MIC) combined with various antibiotics, Figure S6. Effect of LP-SeNPs on *S. aureus* (ATCC 25923).

## Abbreviations

The following abbreviations are used in this manuscript:

ATCC	American Type Culture Collection
CFS	Cell-Free Supernatant
Chem-SeNPs	Chemically synthesized Selenium Nanoparticles
COL	Colistin
DCFH-DA	2′,7′-Dichlorodihydrofluorescein Diacetate
DLS	Dynamic Light Scattering
FICI	Fractional Inhibitory Concentration Index
FTIR	Fourier-Transform Infrared Spectroscopy
HEK	Human Embryonic Kidney
IMP	Imipenem
LP	*Lactiplantibacillus plantarum*
LP-CFS	*Lactiplantibacillus plantarum*-derived Cell Free Supernatant
LP-SeNPs	*Lactiplantibacillus plantarum*-derived Selenium Nanoparticles
MDR	Multidrug-resistant
MIC	Minimum Inhibitory Concentration
MRSA	Methicillin-resistant *Staphylococcus aureus*
NPs	Nanoparticle(s)
ROS	Reactive Oxygen Species
SDS-PAGE	Sodium Dodecyl Sulfate-Polyacrylamide Gel Electrophoresis
SeNP(s)	Selenium Nanoparticle(s)
TET	Tetracycline
TEM	Transmission Electron Microscopy
UV-Vis	Ultraviolet–Visible Spectroscopy
XPS	X-ray Photoelectron Spectroscopy
XRD	X-ray Diffraction
