# Supplementary material for: Biogenic Selenium Nanoparticles from Lactiplantibacillus plantarum as a Potent Antimicrobial Agent Against Methicillin-Resistant Staphylococcus aureus
Source: Pharmaceutics. 2025 Dec 22;18(1):14. doi: 10.3390/pharmaceutics18010014 (PMC12844870; doi:10.3390/pharmaceutics18010014)
Supplement: Supplementary file 1 [file pharmaceutics-18-00014-s001.zip › pharmaceutics-3996836-supplementary.pdf]

Supplementary Materials for

Biogenic Selenium Nanoparticles from *Lactiplantibacillus plantarum* as a Potent Antimicrobial Agent against Methicillin-resistant *Staphylococcus aureus*

Gyeong-min Kim[1], SeCheol Oh[1], and Kwang-sun Kim[1,*]

[1]Department of Chemistry and Chemistry Institute for Functional Materials, Pusan National University, Busan 46241, Korea; eoxhdfud1026@pusan.ac.kr (G.K.); ohs@pusan.ac.kr (S.O.)

*Correspondence: kwangsun.kim@pusan.ac.kr (K.-s. K.)

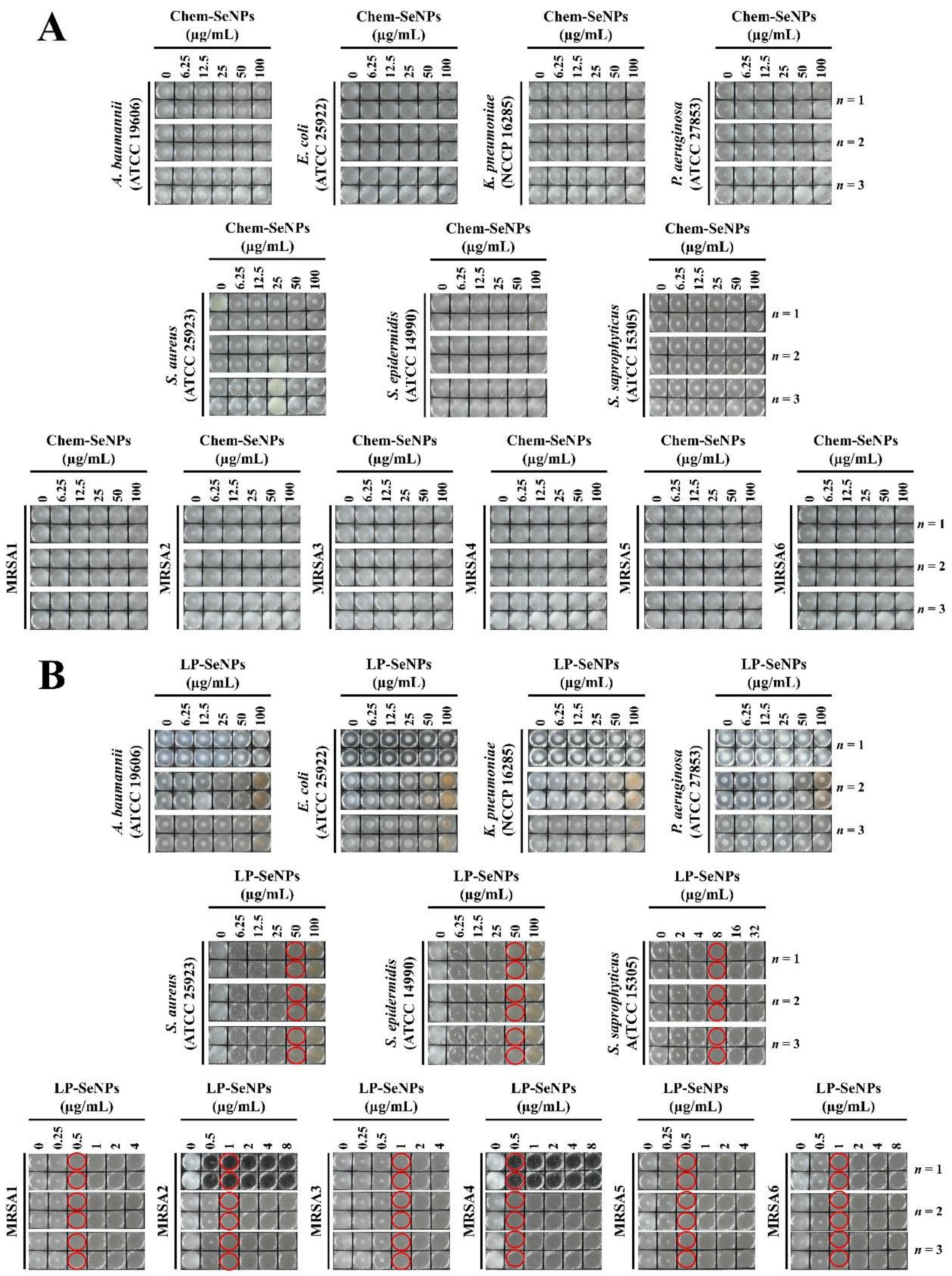

Figure. S1. Images of the MIC assay for Chem-SeNPs and LP-SeNPs are shown. *A. baumannii*, *E. coli*, *K. pneumoniae*, *P. aeruginosa*, *S. aureus*, *S. epidermidis*, *S. saprophyticus*, and clinically isolated MRSA strains were exposed to varying concentrations of (**A**) Chem-SeNPs and (**B**) LP-SeNPs, or left untreated, and incubated in MH broth at 37 °C for 16 h. Photographs of the 96-well plates were taken with a digital camera (Samsung NX200, Suwon, Korea). MIC values, marked by red circles, correspond to those in **Table 2**. Red circles indicate MIC values of LP-SeNPs.

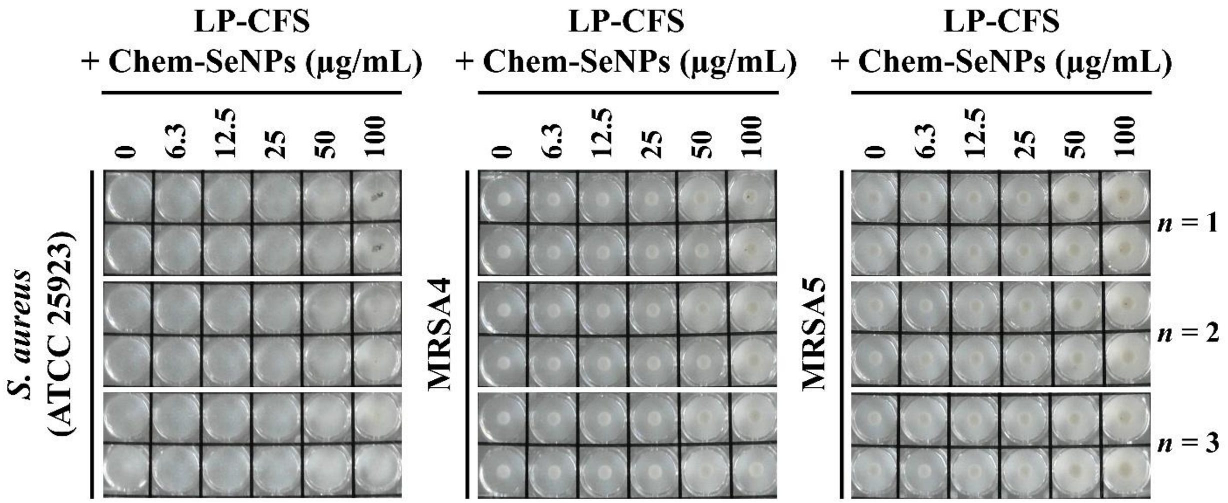

Figure S2. Images of the MIC assay for Chem-SeNPs co-treated with LP-CFS. *S. aureus* (ATCC 25923), MRSA4, and MRSA5 were exposed to various concentrations of Chem-SeNPs (1 mg/mL) were mixed with 1mL of LP-CFS from Section 2.2 and tested for antibacterial activity and subsequently inoculated at a 1:10 ratio for the assay. Photographs of the 96-well plates were captured using a Samsung NX200 digital camera.

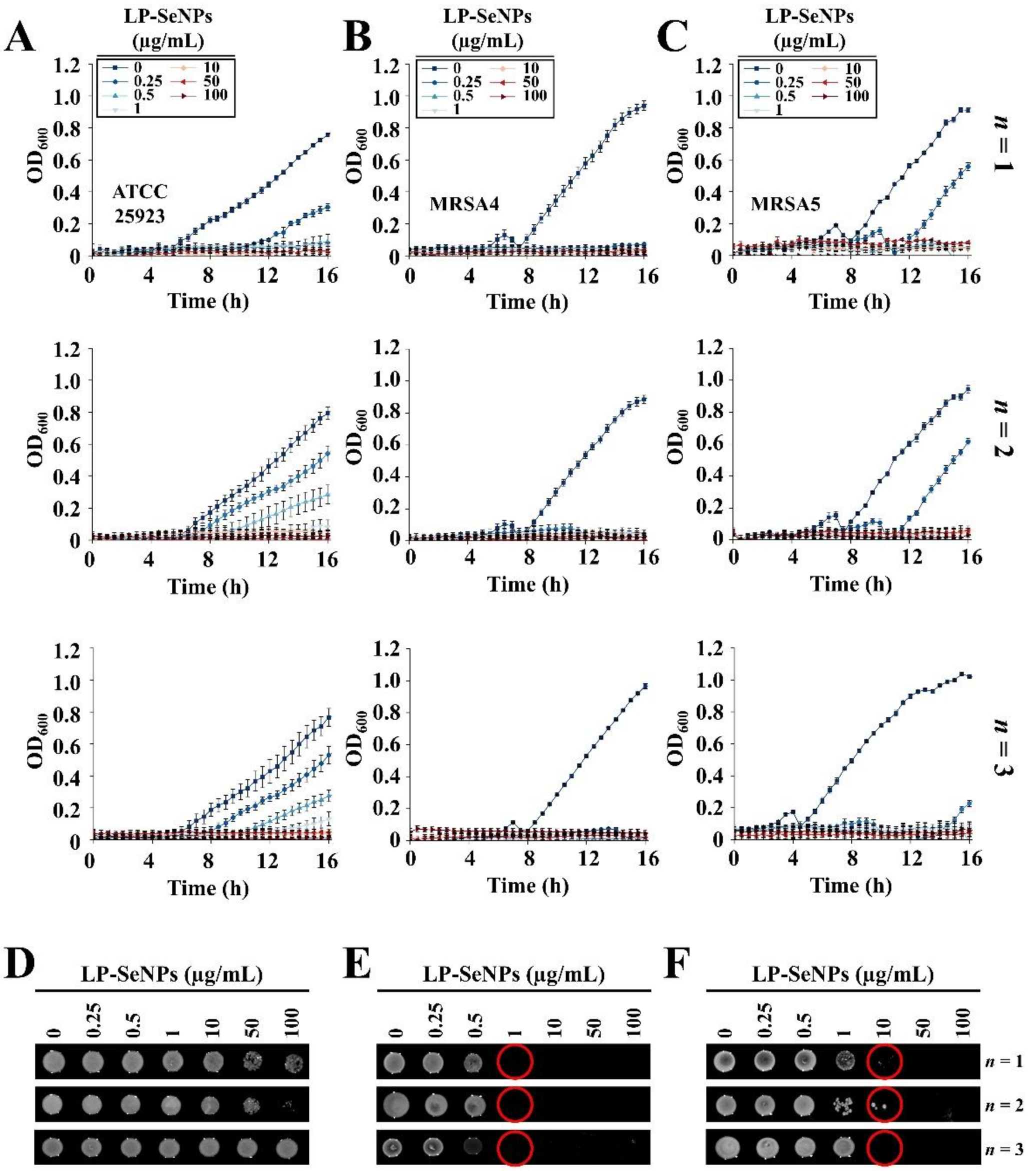

Figure S3. Antibacterial activity of LP-SeNPs. Time–kill kinetics of LP-SeNPs were evaluated against (**A**) *S. aureus* (ATCC 25923), (**B**) MRSA4, and (**C**) MRSA5. Viability assessments corresponding to (**A–C**) are shown in (**D–F**), respectively. Bacterial suspensions were standardized to a 0.5 McFarland standard using a Sensititre™ Nephelometer (Thermo Fisher Scientific, Waltham, MA, USA), diluted 1:1000 in Mueller–Hinton broth (MHB), and treated with various concentrations of LP-SeNPs in 96-well microplates (SPL Life Sciences, Pocheon, Korea). The plates were incubated at 37 °C with shaking at 500 rpm for 16 h, and bacterial growth was monitored by measuring the optical density at 600 nm (OD_{600}) every 30 min using a SPECTROstar® Nano microplate reader (BMG Labtech, Germany). Data were analyzed using MARS software (v3.02 R2, BMG Labtech). For endpoint viability analysis, cultures exposed to LP-SeNPs for 16 h were spot-plated (5 μL) onto LB agar and incubated at 37 °C for an additional 16 h. Colony growth was visualized using a ChemiDoc™ MP Imaging System and quantified with Image Lab™ software (v5.2.1, Bio-Rad, Hercules, CA, USA). Red circles indicate the bactericidal concentration of LP-SeNPs against MRSA4 and MRSA5.

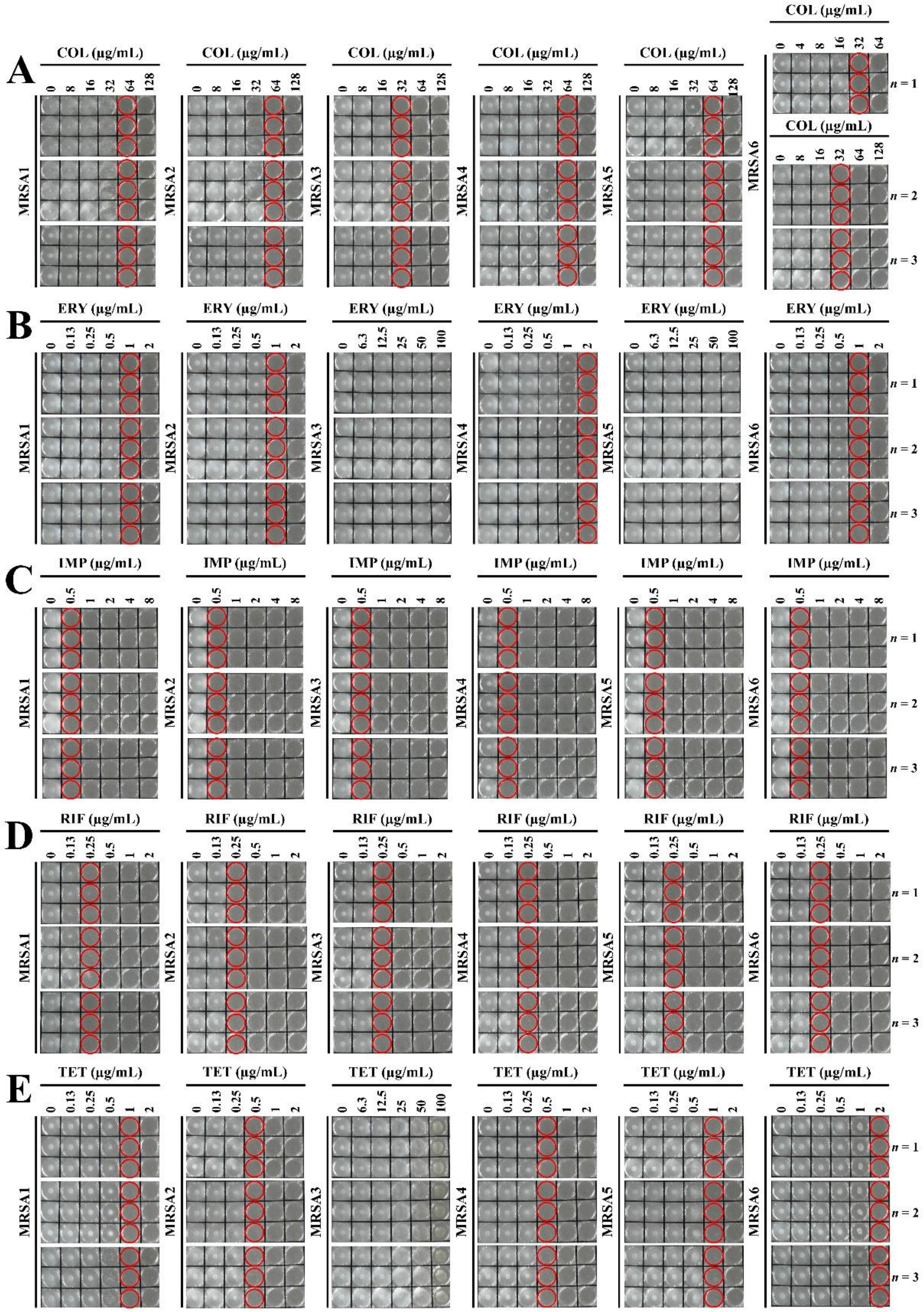

Figure S4. Images of the MIC assay for various antibiotics. Clinical MRSA isolates were exposed to serial concentrations of (**A**) colistin (COL), (**B**) erythromycin (ERY), (**C**) imipenem (IMI), (**D**) rifampicin (RIF), and (**E**) tetracycline (TET), or left untreated, and incubated in MHB at 37 °C for 16 h. Images of the 96-well plates were captured using a digital camera (Samsung NX200). MIC values, indicated by red circles, correspond to those listed in **Table 3**. Red circles indicate MIC values of individual antibiotics.

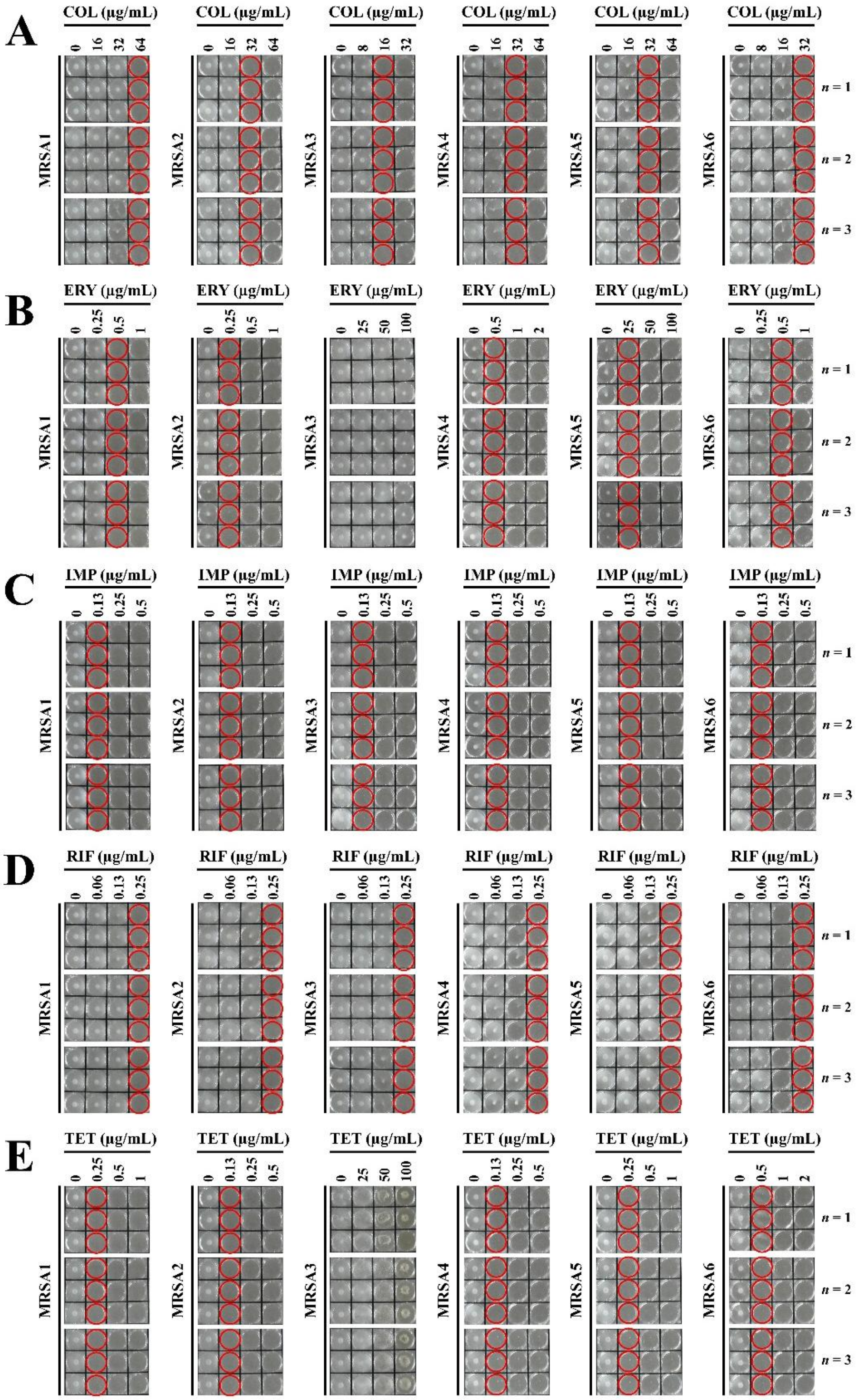

Figure S5. Synergistic effects of LP-SeNPs (¼ MIC) combined with various antibiotics. Clinical MRSA isolates were exposed to varying concentrations of (**A**) colistin (COL), (**B**) erythromycin (ERY), (**C**) imipenem (IMI), (**D**) rifampicin (RIF), and (**E**) tetracycline (TET) with ¼ MIC of LP-SeNPs were incubated in MHB at 37 °C for 16 h. Images of the 96-well plates were captured using a digital camera (Samsung NX200). MIC values, marked by red circles, correspond to those listed in **Table 4**. Red circles indicate MIC values of individual antibiotics in combination treatments.

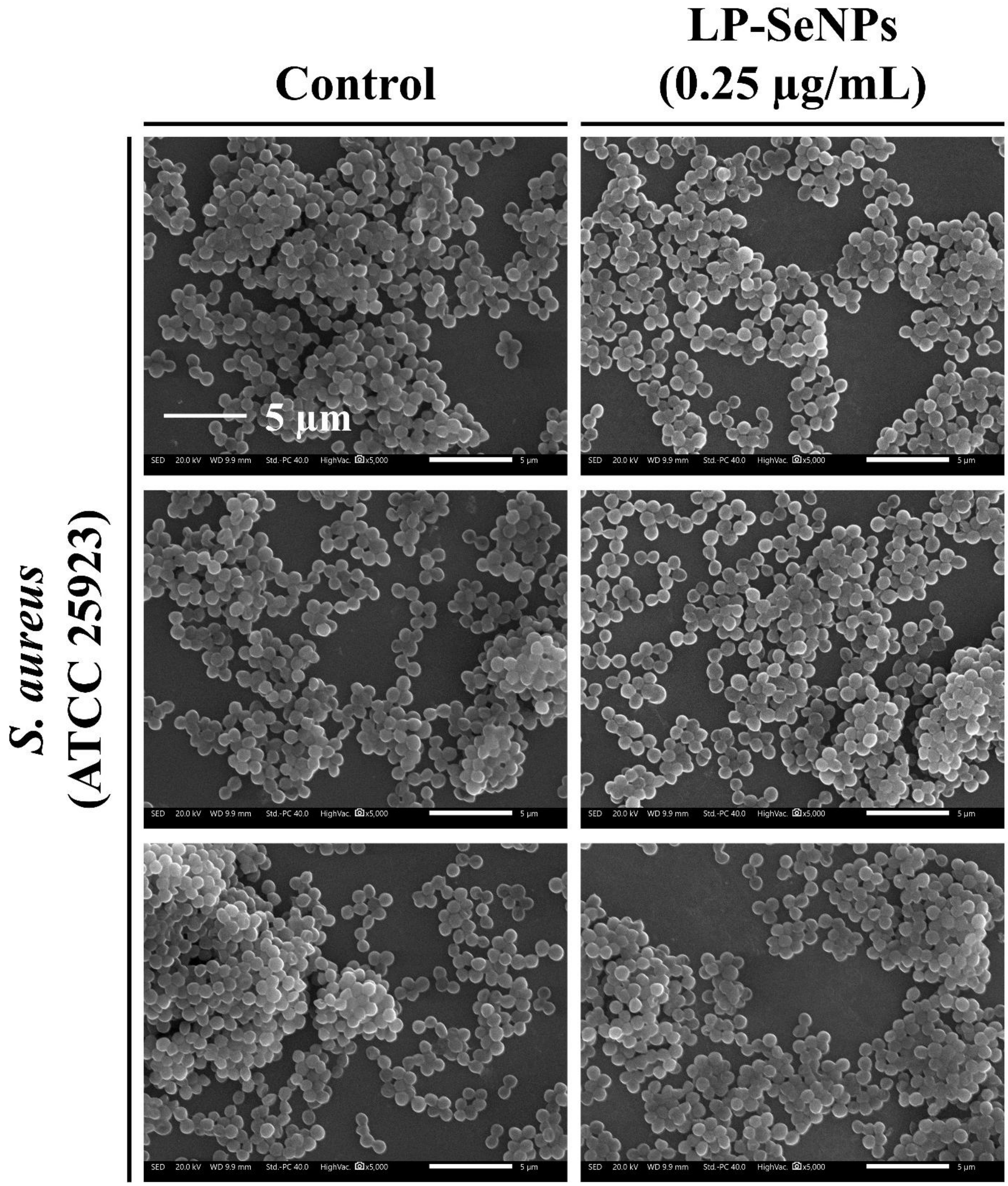

Figure S6. Effect of LP-SeNPs on *S. aureus* (ATCC 25923). SEM images illustrate the morphological changes in *S. aureus* cells after 4 h of incubation with and without LP-SeNPs (0.25 μg/mL; ½ MIC).
